# The Interplay of Cholesterol and Ligand Binding in *h*TSPO from Classical Molecular Dynamics Simulations

**DOI:** 10.3390/molecules26051250

**Published:** 2021-02-26

**Authors:** Hien T. T. Lai, Alejandro Giorgetti, Giulia Rossetti, Toan T. Nguyen, Paolo Carloni, Agata Kranjc

**Affiliations:** 1 VNU Key Laboratory for Multiscale Simulation of Complex Systems, VNU University of Science, Vietnam National University, Hanoi 11416, Vietnam; laithithuhien_t60@hus.edu.vn; 2 Institute of Neuroscience and Medicine (INM-9), Forschungszentrum Jülich, D-52425 Jülich, Germany; alejandro.giorgetti@univr.it (A.G.); g.rossetti@fz-juelich.de (G.R.); p.carloni@fz-juelich.de (P.C.); 3 Institute for Advanced Simulation (IAS-5), Forschungszentrum Jülich, D-52425 Jülich, Germany; 4 Department of Biotechnology, University of Verona, Strada Le Grazie 15, 37134 Verona, Italy; 5 Jülich Supercomputing Center (JSC), Forschungszentrum Jülich, 52428 Jülich, Germany; 6 University Hospital Aachen, RWTH Aachen University, 52078 Aachen, Germany; 7 Department of Physics, RWTH Aachen University, 52078 Aachen, Germany; 8 JARA-BRAIN Institute “Molecular Neuroscience and Neuroimaging” INM-11, Forschungszentrum Jülich, 52428 Jülich, Germany; 9 Laboratoire de Biochimie Théorique, UPR 9080 CNRS, Université de Paris, 13 rue Pierre et Marie Curie, F-75005 Paris, France; 10 Institut de Biologie Physico-Chimique-Fondation Edmond de Rotschild, PSL Research University, 75005 Paris, France

**Keywords:** *h*TSPO, PK11195, cholesterol, homology modeling, molecular dynamics (MD) simulation

## Abstract

The translocator protein (TSPO) is a 18kDa transmembrane protein, ubiquitously present in human mitochondria. It is overexpressed in tumor cells and at the sites of neuroinflammation, thus representing an important biomarker, as well as a promising drug target. In mammalian TSPO, there are cholesterol–binding motifs, as well as a binding cavity able to accommodate different chemical compounds. Given the lack of structural information for the human protein, we built a model of human (*h*) TSPO in the apo state and in complex with PK11195, a molecule routinely used in positron emission tomography (PET) for imaging of neuroinflammatory sites. To better understand the interactions of PK11195 and cholesterol with this pharmacologically relevant protein, we ran molecular dynamics simulations of the apo and holo proteins embedded in a model membrane. We found that: (i) PK11195 stabilizes *h*TSPO structural fold; (ii) PK11195 might enter in the binding site through transmembrane helices I and II of *h*TSPO; (iii) PK11195 reduces the frequency of cholesterol binding to the lower, N–terminal part of *h*TSPO in the inner membrane leaflet, while this impact is less pronounced for the upper, C–terminal part in the outer membrane leaflet, where the ligand binding site is located; (iv) very interestingly, cholesterol most frequently binds *simultaneously* to the so-called CRAC and CARC regions in TM V in the free form (residues L150–X–Y152–X(3)–R156 and R135–X(2)–Y138–X(2)–L141, respectively). However, when the protein is in complex with PK11195, cholesterol binds equally frequently to the CRAC–resembling motif that we observed in TM I (residues L17–X(2)–F20–X(3)–R24) and to CRAC in TM V. We expect that the CRAC–like motif in TM I will be of interest in future experimental investigations. Thus, our MD simulations provide insight into the structural features of *h*TSPO and the previously unknown interplay between PK11195 and cholesterol interactions with this pharmacologically relevant protein.

## 1. Introduction

The translocator protein (TSPO) is a transmembrane protein (18kDa), evolutionary conserved and expressed in different organisms, from bacteria to humans [1]. Its biological functions are conserved throughout the phylogenetic spectrum, like tetrapyrrole biosynthesis and/or sterol metabolism [1,2]. Indeed, the bacterial TSPO homology in *Rhodobacter sphaeroides* can be functionally replaced by rat TSPO [3], despite that these proteins share only about 30% sequence identity. The human protein (*h*TSPO) is expressed in all tissues and located in the outer mitochondrial membrane [4,5]. Its highest expression levels are found in steroid-synthesizing cells of endocrine organs indicating that it may play an important role in steroid synthesis from cholesterol [6]. Mammalian TSPO binds cholesterol with high affinity by the cholesterol recognition/interaction amino acid consensus (CRAC) motif (residues 150–156) [7,8]. This motif is preceded by a short three amino acids sequence L144–A145–F146 (LAF) that is highly conserved in mammalian TSPO. In the experiment with the *Rs*TSPO mutant, where the three amino acids (A136–T137–A138) preceding the CRAC region were replaced by mammalian LAF sequence, it was shown that the LAF motif greatly increased the binding affinity for cholesterol with respect to the original bacterial sequence [9]. An additional binding prediction sequence for cholesterol was found in TSPO—the inverse version of CRAC, the CARC motif (residues 135–141) [10]. While in the case of the nicotine acetylcholine receptor, functional studies clearly show that a substitution of a specific amino acid in CARC slows the kinetics of cholesterol binding, in the case of TSPO, it is not yet known whether CARC binds cholesterol as well [11]. In addition, TSPO has been proposed to play an important role in other cellular processes like porphyrin transport [12,13], mitochondrial respiration [4,14], and immunomodulation [15].

TSPO expression is highly upregulated in cancer and at the sites of neuroinflammation processes in cerebral ischemia, Alzheimer’s, Parkinson’s, and Huntington’s diseases, and multiple sclerosis (reviewed in [16]). In addition, a human single nucleotide polymorphism of TSPO (A147T) is associated with different psychiatric disorders, like bipolar disorder, anxiety, and panic attacks [17,18,19], along with cancer. Thus, TSPO is an interesting target for the development of diagnostic and therapeutic ligands [16,20]. TSPO is overexpressed in the outer mitochondrial membrane of activated microglia [21,22,23] and reactive astrocytes [24]. Chronic activation of microglia leads to the release of neurotrophic and proinflammatory factors that are neurotoxic and cause neuronal damage and neurodegeneration [25,26,27]. The microglial activation is imaged in human brain in vivo by positron emission tomography (PET) of TSPO radiolabeled ligands. PK11195 (1-(2-chlorophenyl)-*N*-methyl-*N*-(1-methylpropyl)-3-isoquinolinecarboxamide) is one of the most commonly used, high affinity TSPO ligands for studying the diagnostics and treatment of brain inflammation and of other inflammatory diseases [28].

Second- (PRB28, PBR06, DAA1106) and third- (ER176 and GE-180) generation ligands have been developed (reviewed in [29,30]). PRB28, PBR06, and DAA1106 have higher binding affinity for *h*TSPO than PK11195. They provide a better signal-to-typical positron emission tomography (PET) noise ratio. ER176 and GE-180 are being developed to overcome differences in binding affinities for the WT or the A147T mutant *h*TSPO. This mutant emerges in 30% of Caucasians, 25% of Africans, 4% of Japanese, and 2% of Han Chinese according to the Hapmap database (http://hapmap.ncbi.nlm.nih.gov (accessed on 25 January 2020)). It binds PRB28 [31], PBR06 [32], and FEPPA [33]. This consequently leads to the lower PET signal intensity and can provide misleading results (absence of neuroinflammation) for the carrier of the A147T mutation.

TSPO folds into a bundle of five transmembrane (TM) helices and a short extramembrane helix placed in the cytoplasmic loop between helices TM I and TM II [34,35,36]. Bacterial and mouse TSPO can exist as monomers, dimers, and other oligomers as shown by experiments [34,35,37,38]. Different computational molecular modeling studies were carried out to further shed light on the dimerization of mouse and bacterial TSPO and on how ligands (small chemical compounds, porphyrin, cholesterol) interact with dimers or influence their stability [39,40,41,42]. In contrast, the oligomerization state of *h*TSPO has not been established. An in vitro study showed that it can adopt dimeric or trimeric forms under the inflammation conditions reproduced by high concentrations of reactive oxygen species (ROSs) [43]. Different experimental TSPO structures have been solved to date, notably the NMR structure of mouse TSPO (*Mo*TSPO, PDB ID: 2MGY [36]) and the X-ray structures of *Rhodobacter sphaeroides* (*Rs*TSPO, PDB ID: 4UC1 [35]) and *Bacillus cereus* (*Bc*TSPO, PDB ID: 4RYI [34]) TSPOs.

The experimental structure of *h*TSPO has not yet been solved. Here, we built a monomeric structural model of *h*TSPO, alone and in complex with PK11195 by homology modeling and docking, and we ran molecular dynamics (MD) simulations of the protein in a lipid environment. We analyzed in detail the interactions of PK11195 and cholesterol with *h*TSPO and how PK11195 alters cholesterol interactions with this protein. Our structural model and results can be a valuable source for future studies of *h*TSPO and its interactions with cholesterol and/or other pharmacological ligands.

## 2. Results and Discussion

### 2.1. The *h*TSPO Structural Model

**Figure 1:**
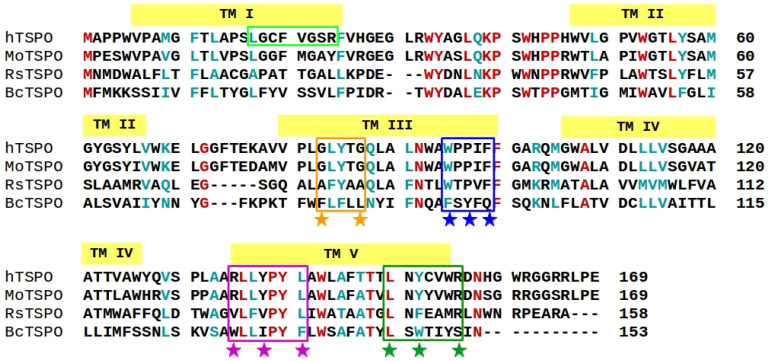
Multiple sequence alignment of TSPO from different organisms: human TSPO (*h*TSPO), *Mus musculus* (*Mo*TSPO) [36], *Rhodobacter sphaeroides* (*Rs*TSPO) [35], and *Bacillus cereus* (*Bc*TSPO) [34]. For the last three proteins, experimental structural information is available. Semi-conserved positions with more than 50% consensus according to ClustalO [46] are highlighted in cyan, while highly conserved positions with more than 90% consensus are shown in red. The oligomerization motif G83XXXG87 is indicated by the orange stars and rectangle. The W95XPXF99 motif is depicted with the blue stars and rectangle. The cholesterol-binding motif CRAC and its “mirror code” CARC are marked by dark green and violet rectangles and stars, respectively. The CRAC-like motif in TM I is highlighted by the light green rectangle.

The sequence of the *h*TSPO was aligned with those of *Rs*TSPO, *Bc*TSPO, and *Mo*TSPO (the sequence identity is of 29%, 24%, and 81%, respectively; Figure 1). We decided to use *Rs*TSPO (PDB code: 4UC1 [35]) as a template in structural modeling of the human translocator protein [44,45].

Indeed, the latter is currently the best template choice in comparative modeling of mammalian TSPOs [40,41] for the following reasons: (i) the structural folds of the TSPOs on passing from the bacterial to the mammalian proteins are conserved [34,35,36]; (ii) the NMR *Mo*TSPO structure is affected by the ionic detergents used for the purification during the measurements [36]; as a result, the positions of highly conserved amino acids and of the transmembrane helices are altered [40]; (iii) the *Rs*TSPO crystal structure was resolved at a relatively good resolution (1.8 Å).

**Figure 2:**
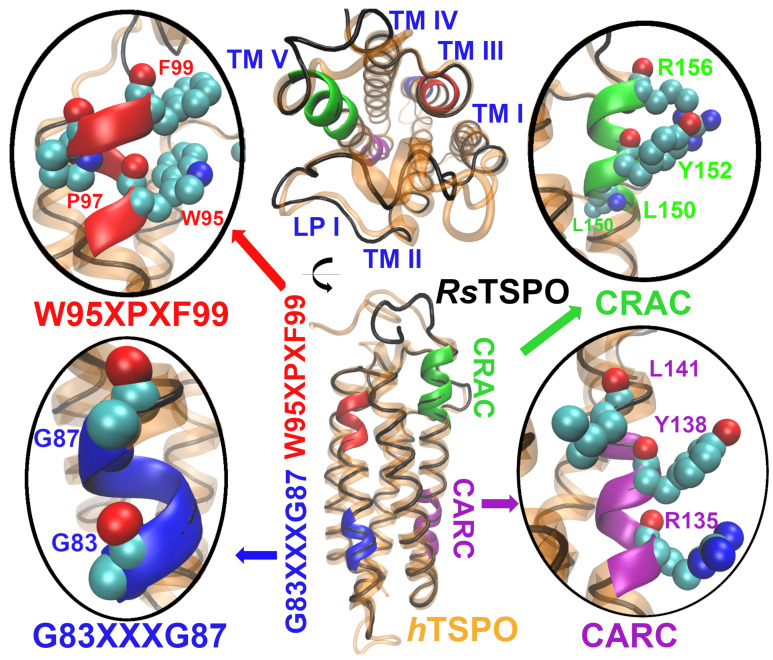
Top and side view of the structural model of the *h*TSPO monomer (orange cartoon representation) aligned with the *Rs*TSPO template (black tube representation). Four important functional regions are highlighted: cholesterol recognition/interaction amino acid consensus region (CRAC, in green color) and reverse region of the CRAC (CARC, in purple color), both involved in cholesterol binding, the G83XXXG87 motif (blue color) relevant for monomer–monomer interactions, and the W95XPXF99 motif (shown in red color) important for ligands binding. The figure was prepared with the VMD program [50].

We modeled the *h*TSPO based on the canonical *h*TSPO sequence reported in the UniProtKB database, Entry P30536 [47,48] (https://www.uniprot.org/uniprot/P30536 (accessed on 1 February 2019)). The model contains five transmembrane helices (TMs) arranged in the clockwise order TM I–TM II–TM V–TM IV–TM III. The four loops are located either in the cytoplasm, LP I and LP III, or in the cytosol, LP II and LP IV, respectively (Figure 2). LP I is composed of residues V26–H46 (Figure 1), and it was earlier suggested to play a role as the gate of the *h*TSPO ligand binding pocket [49]. Almost half of the residues composing LP I are fully conserved between *h*TSPO and *Rs*TSPO (Figure 1), which has a short α-helix in the middle of its loop. LP I in the *Mo*TSPO structure is shorter (10 residues) than in the bacterial TSPO structures (19 residues), and it lacks this α-helix.

Different structurally and/or functionally important motifs are conserved among *Rs*TSPO and *h*TSPO:

(i) An important feature of the mammalian TSPO is its capability of binding cholesterol molecules [7]. Two motifs are involved in cholesterol binding: the cholesterol recognition/interaction amino acid consensus motif (CRAC) represented as L150–X–Y152–X(3)–R156 (Figure 1, dark green rectangle) [7] and the reverse region of CRAC (named CARC) described as R135–X(2)–Y138–X(2)–L141 (Figure 1, purple rectangle) [7,10]. One helical turn before CRAC, there is a short sequence L144–A145–F146 that enhances the binding affinity of mammalian TSPO for cholesterol [9]. Even though cholesterol is absent in bacterial membranes, the CRAC motif is conserved in *Rs*TSPO (Figure 1, dark green rectangle). This is most probably in order to accommodate hopanoids, which have a structure and function similar to that of cholesterol in higher organisms [51].

In order to interact with cholesterol, side chains of the key CRAC, CARC, and LAF residues need to face the membrane. In our model, CRAC Y152 and R156 side chains are membrane exposed, while L150 is oriented towards the ligand binding cavity in the center of the TSPO (Figure 2). Different mutagenesis studies showed the importance of Y152 and of R156 for cholesterol binding. If one of these two residues is mutated to serine or leucine, respectively, the binding of cholesterol is abolished [7,8]. CARC, in contrast to CRAC, is not conserved in *Rs*TSPO (Figure 1, purple box). However, in our *h*TSPO model, all key residues from this motif, R135, Y138, and L141, are membrane exposed and can interact with cholesterol (Figure 2). Among the LAF residues, L144 is facing the interior of the protein in our model, while A145 and F146 are membrane exposed and available for cholesterol binding.

(ii) The G83XXXG87 motif from *h*TSPO (Figure 1, orange rectangle) coincides with the A75XXXA79 motif in *Rs*TSPO. These motifs represent widespread helix–helix interactions across different membrane proteins [52,53,54,55,56]. Both motifs are located in the third TM domain (TM III) of the respective proteins and represent a binding interface for TSPO monomer–monomer interactions. The G83 and G87 residues are exposed to the membrane in our *h*TSPO model and can interact with the second monomer (Figure 2).

(iii) The W95XPXF99 motif is fully conserved among human, mouse, and *Rs*TSPO, while the *Bc*TSPO sequence differs significantly in this region (Figure 1, blue rectangle). This motif is conserved also in other prokaryotic and eukaryotic TSPO sequences [40,57], and it was suggested to play a role in oligomerization processes [49], as well as in ligand binding [34,58]. In the *Mo*TSPO experimental structure, W95 points into the binding cavity and F99 is oriented toward the membrane, whereas in the *Rs*TSPO structure, both residues point into the binding pocket. In the *Bc*TSPO–PK11195 complex, residues F90 and Q94, which correspond to W95 and F99 in mammalian TSPO, respectively, interact with PK11195. This is also the case for our *h*TSPO model (Figure 2).

### 2.2. PK11195 Interactions with the *h*TSPO Model

We docked PK11195 to our *h*TSPO model. Two 3D structures of the TSPO in complex with PK11195 exist, the NMR structure of *Mo*TSPO [36] and the X-ray structure of *Bc*TSPO [34]. The ligand binding cavity has the same location in both proteins, but PK11195 adopts different binding poses. In our studies, we used the *h*TSPO–PK11195 complex where the ligand binding pose was similar to the one observed in the *Bc*TSPO, since currently, there are no experimental structures available for the *Rs*TSPO–PK11195 complex, which is a template of our model. Other docking poses differed from the one used as the starting configuration for the MD simulations (Figure S1), showing a small degree of convergence. Indeed, these poses are already cluster representatives, since AutodockVina [59], used here, does the clustering automatically.

However, many residues crucial for PK11195 binding in the *Bc*TSPO crystal structure are fully conserved in *Rs*TSPO, like Y32(31), P42(41), W51(50), N87(84), W138(135), A142(139), and L145(142) (numbering is for *Bc*TSPO and in parentheses for *Rs*TSPO) (Figure 1). Furthermore, the main structural differences between *Bc* and the *Rs*TSPOs appear at the monomer–monomer interface in the dimer structure and not in the ligand binding pocket [49].

The binding cavity in our model is lined with residues belonging to four TM domains and to LP I: G18, C19, V21, G22, F25 (in TM I), Y34, H43 (in LP I), H46, L49, G50, W53 (in TM II), N92, W95, P96, F99, F100 (in TM III), W143, T147, L150 (in TM V). The residues L49, W53, W95, W143, A147, and L150 are involved in the binding of PK11195 in both the *Mo* and *Bc*TSPOs experimental structures [34,36]. Beside these residues, others bind PK11195 in *Mo* and *Bc*TSPO, but they are specific to each structure. One third of the binding residues in our model are fully conserved among mammalian [60] and other prokaryotic and eukaryotic species [57], like Y34, W53, N92, W143, L150, and A/T147 (Figure 1, for more complete alignments see [57,60]). It was shown that either threonine or alanine at position 147 has no impact on PK11195 binding to the TSPO since it binds to both polymorphs with the same binding affinity, in contrast to other radioligands, which bind with significantly smaller binding affinity to the protein with threonine [31,60,61].

**Figure 3:**
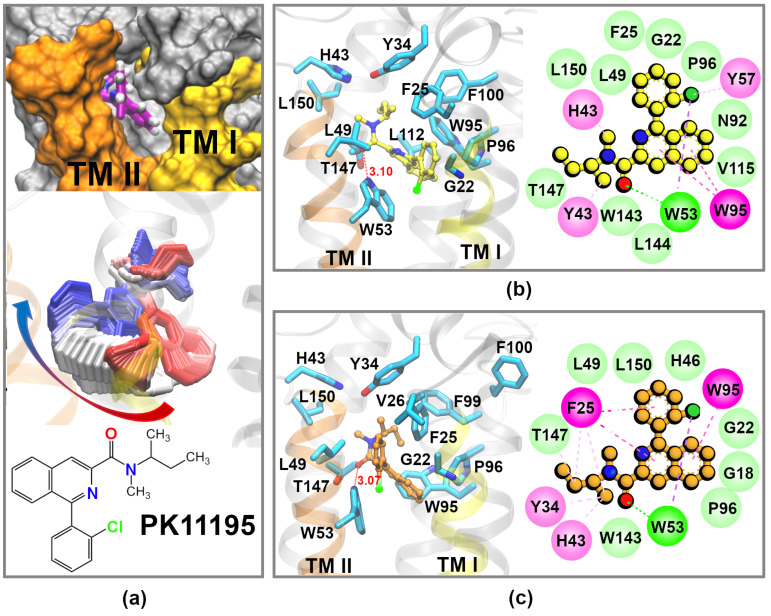
PK11195 binding interactions with the *h*TSPO model. (**a**) Top: Open access to the binding pocket between TM I and TM II of the *h*TSPO structural model that persists throughout the MD simulation. We do not observe other openings, nor the change in the LP I conformation. Center: PK11195 moves in the binding site of the *h*TSPO model from its initial state observed in the first 450 ns (red color) to the new pose (blue color, 450–1000 ns). The image of every hundredth frame is shown smoothed with a five frame window. Bottom: Chemical formula of PK11195. (**b**,**c**) 3D and 2D representations of the PK11195 binding pocket during the first 450 ns (top) and after the ligand movement, from 450 ns till the end of the MD run (bottom). 3D plots show PK11195 (yellow and orange balls and sticksrepresentation for 0–450 ns and for 450–1000 ns, respectively) and the residues binding it for more than 90% of the simulation time; the backbone and hydrogen atoms were omitted for clarity reasons. F100 was kept in (**c**), despite that it does not bind PK11195 anymore, to show the change in its side chain conformation. The most constant interactions, formed for more than 75% of the simulation time between PK11195 and the *h*TSPO model, are shown in the 2D plots obtained by the Discovery tool [62]. Legend: green circles—hydrogen bonds, light green circles—VdW interactions, light pink circles—π-alkyl, and dark pink circles—π-π interactions.

Next, we ran a 1 μs long MD simulation and analyzed the interactions between our *h*TSPO model and the PK11195 ligand. Additional MD simulation replicas of 450 ns were later run for holo and apo *h*TSPOs (two for each protein), and the results are reported in the Supplementary Materials. During the MD simulation, at around 450 ns, we observed quite sudden movement of the PK11195 chlorophenyl-*N*-isoquinoline part towards different binding pose, while the alkyl part of PK11195 remained at its initial position. The rings horizontally slide in a way that the chlorophenyl ring, which initially faces TM I, is placed between TM I and TM II, closer to the latter helix (Figure 3a, center, and Figure S2). This movement indicates that the initial binding pose may not be optimal and/or that the TSPO binding pocket possesses certain plasticity allowing for different binding poses of the ligand. Interestingly, the pose of PK11195 after 450 ns was similar to one docking position (Figure S1, pose 5; the RMSD between the two poses is 1.8 Å). We compared the binding pose that PK11195 adopts during the first 450 ns (Figure 3b) with the one it takes up after the movement (Figure 3c). We observed that residues binding constantly PK11195 during the whole length of the MD run are: G22, F25 (in TM I), Y34, H43 (in LP I), L49, W53 (in TM II), W95, P96 (in TM III), and T147, L150 (in TM V). Residues Y34, W53, W95, A/T147, and L150 are well conserved among TSPOs from different species, while G22, F25, and L49 are semi-conserved [57]; this indicates their importance for the structure and/or function of the protein.

The F25 side chain is initially facing towards the membrane, later it moves inside the binding pocket, establishing the π-stacking and hydrophobic interactions with F100, Y34, and PK11195. In the *Mo*TSPO (PDB code: 2MGY [36]) and *Bc*TSPO (PDB code: 4RYI [34]) structures, this residue points out of the binding site in the same orientation as it does in our model at the beginning of the MD simulation.

In our model, Y34 forms hydrophobic interactions with PK11195 throughout the MD simulation. This residue is fully conserved among TSPOs from different species, from mammalians to bacteria [57]. However, in the crystal structure of *Bc*TSPO–PK11195, this residue binds PK11195, while in the *Mo*TSPO–PK11195 complex, it faces the cytosol. Despite this ambiguity in the TSPO–PK11195 experimental structures, our result is in very good agreement with mutational studies showing that mutations Y34F, Y34F/F100A, and Y34F/F99A cause a large decrease in the binding affinity for PK11195 with respect to the WT TSPO [58]. These results indicate that the aromatic phenyl rings are crucial at this place for PK11195 binding.

A stable hydrogen bond (H-bond) is formed between the W53 indole amino group and the carbonyl-oxygen atom of PK11195. The H-bond between W53 and PK11195 was observed also in the *Bc*TSPO–PK11195 crystal structure [34], but not in the *Mo*TSPO–PK11195 NMR structure, which lacks any H-bond interaction [36]. Another H-bond observed in the *Bc*TSPO–PK11195 structure was formed between W143 and the PK11195 ligand [34]. In our model, this H-bond is formed occasionally during the first 450 ns; after this time, W143 constantly interacts with PK11195 through VdW interactions.

PK11195 is additionally bound through VdW, hydrophobic, or stacking interactions by H43, L49, P96, W95, T147, and L150. Some of these residues interact with PK11195 also in the *Bc*TSPO structure (F90, S91, A142, and L145, respectively) and in the *Mo*TSPO structure.

In addition to all the above described residues, F100 and L112 steadily bind PK11195 during the first 450 ns. At this time, the ligand moves to a new binding pose, and these two interactions are lost; however, the interactions with V26 and F99 are established (Figure 3b,c). Interestingly, the F100 side chain flips out of the binding pocket at around 720 ns (Figure 3c). This residue is oriented towards the binding site in *Bc*TSPO, as it is in our model at the beginning of the MD simulations, while in *Mo*TSPO, it is facing the membrane like in our model at the end of the MD run. According to our results, we suggest that F100 spontaneously changes its position from inward to outward of the binding pocket and that the experimental structures captured it in one of these two different conformations.

The spontaneous change that we observe in the orientation of the F100 side chain may indicate that it is involved in placing the ligand inside the binding pocket, but not crucial for its binding, and that this role is left to F99 and Y34, as suggested by [58]. Deeper studies will of course need to be done to explore in detail the exact role of these three residues.

Different hypothesis were made in the literature about the possible binding pathways for PK11195: one suggested that the ligand enters the binding pocket from the cytosol and that LP I plays a role of the gate [49,63]; the second one proposes binding between the crevices in TM helices [34,63,64].

In the present work, we observed the opening between TM I and TM II of our *h*TSPO–PK11195 model, which during the whole simulation time gave direct access to the binding pocket through the membrane (Figure 3a). Furthermore, PK11195 adopts a position where its Cl-phenyl ring is placed in this crevice, enveloped by C19, G22, F25, and V26 from TM I and by H46, L49, and W53 from TM II. In contrast, in the apo *h*TSPO model, the TM I and TM II helices maintain the closed position, without any openings, during the full length of the MD simulation (Figure S3).

Furthermore, we noted that LP I is stabilized through a patch of interactions: cation-π interactions formed between K39 (LP I) and Y34 (LP I) and the stacking interactions between Y34, F25 (TM I), F99, F100 (TM III), and PK11195. These interactions prevent—within the time scale of our simulation—LP I from moving in a way to open the access to the binding site from the top of the protein and therefore from the cytosol. This result is consistent with the previous study [64]. However, we cannot exclude that on a longer time scale, LP I is able to perform larger movements, as was proposed by [63].

### 2.3. PK11195 Stabilizes *h*TSPO Structural Fold

**Figure 4:**
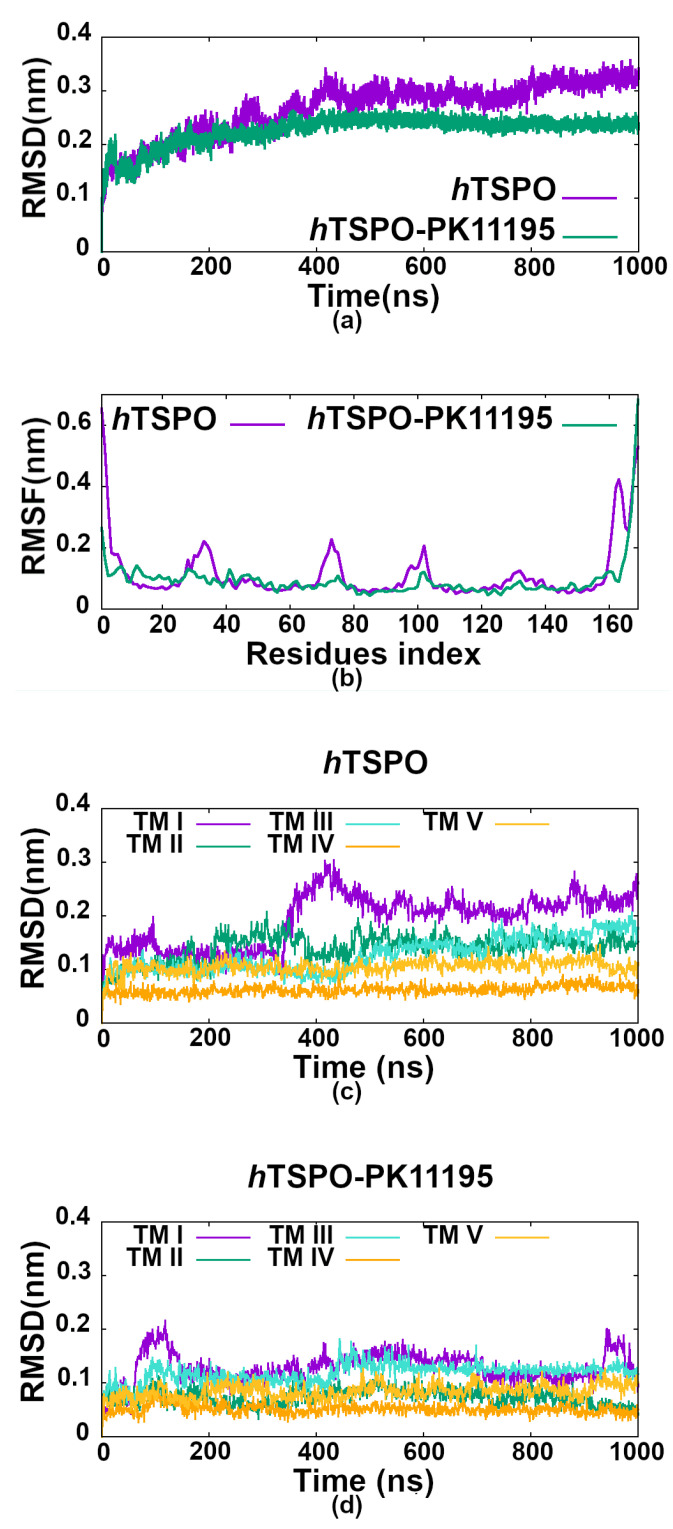
(**a**) Evolution of the root mean squared deviation (RMSD) values of the apo (violet graph) and holo (green graph) *h*TSPOs during the 1 μs long MD simulation. RMSD values were calculated for the backbone atoms of residues W5 to N158, excluding the N– and C–termini and H atoms. (**b**) Root mean squared fluctuations (RMSF) of the Cα atoms in the apo (violet graph) and holo (green graph) *h*TSPOs. RMSF values were calculated for equilibrated proteins (in the MD simulation range of 400 ns–1 μs). (**c**,**d**) RMSD values for each of the five transmembrane helices (TM I–TM V) in the apo (*h*TSPO) and holo (*h*TSPO–PK11195) proteins, respectively.

**Figure 5:**
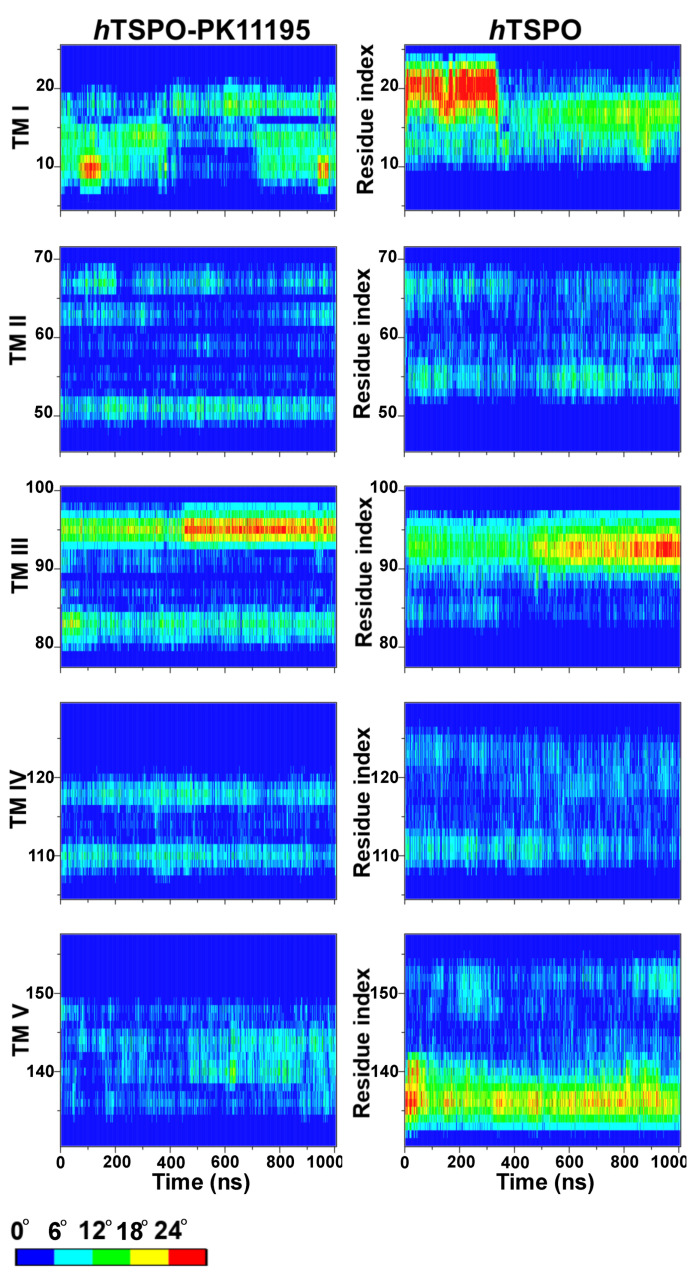
Analysis of the flexibility of each TM domain (TM I–TM V) in *h*TSPO–PK11195 and *h*TSPO structural models by means of Bendix [67]. y-axis: residue index number corresponding to the residues composing individual TM domain; x-axis: simulation time. The color scale indicates changes in helix angle/bending during the MD simulations, from blue: <6${}^{\circ }$ to red: >24${}^{\circ }$.

The apo and holo *h*TSPO structural models were embedded into the membrane and evaluated by a 1 μs long MD simulation. We evaluated and compared the structural stability of the apo and holo *h*TSPOs by calculating the root mean squared deviations (RMSD) of backbone atoms, the root mean squared fluctuations (RMSF) of Cα atoms (Figure 4), and the helices’ flexibility (Figure 5).

**Figure 6:**
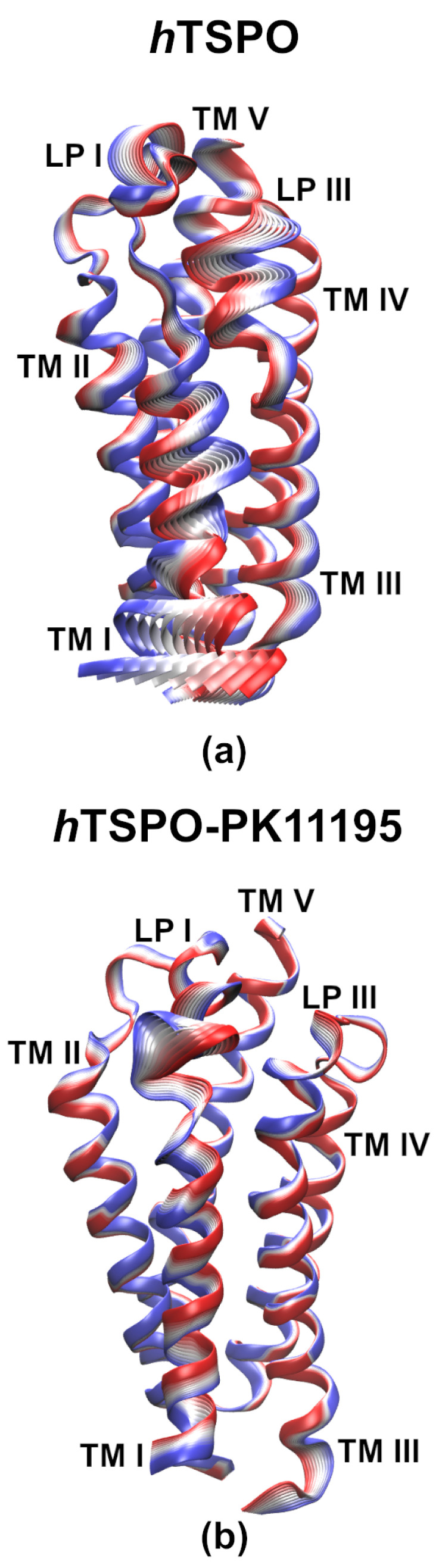
The principal component analysis of the (**a**) *h*TSPO and (**b**) *h*TSPO–PK11195 models showing the flexible parts of the protein. The image of every hundredth frame is shown, spanning from the beginning (red color) to the end (blue color) of the MD simulation.

The RMSD of apo *h*TSPO fluctuates more than that of the holo protein (Figure 4a); however, both systems reach a plateau at around 400 ns. The RMSD fluctuations in the apo protein (from 400 ns on) are principally due to the bending of the TM I helix and due to the flexibility of the loops LP I, II, and III. In contrast, the RMSD of the *h*TSPO–PK11195 complex is lower than for the apo protein and becomes steady from around 400 ns on, indicating that PK11195 stabilizes the *h*TSPO structural model. The higher structural stability of the holo protein can be observed as well from the principal component analysis (PCA) (Figure 6). These results are in line with experimental data for *Mo*TSPO, where the interactions with its cognate ligand PK11195 stabilize its structural fold [36,65]. Similar observations were obtained for *Rs*TSPO, which also showed an important flexibility, especially around the ligand binding site [35,66]. It was shown that the quality of *Rs*TSPO crystals was significantly improved by adding cholesterol and PK11195 to the crystallization medium [35], suggesting that PK11195 can have a positive impact also on the *Rs*TSPO and not only on *Mo*TSPO structural stability.

The RMSD values of backbone atoms for each transmembrane helix (Figure 4c,d) clearly show that PK11195 increases the stability of TM I and TM II, while TM IV and TM V are stable regardless of the absence/presence of the ligand. In both—apo and holo—proteins TM I is the most flexible helix and TM IV the most stable one.

The RMSD for TM I in the apo system has two plateau levels indicating a conformational change. Indeed, there is a kink in the α-helix due to the P15 residue (Figure 5, TM I in *h*TSPO). Helix kinks are a common feature of long α-helices, which are frequent in transmembrane proteins, and proline residues are strongly associated with the helix being kinked [68,69]. TM I becomes straight at around 350 ns. The alteration between the kinked and straight form of TM I is the reason for the RMSD change and also for the higher RMSD values with respect to the other helices in our model (Figure 4c). TM III (P96–P97) and TM V (P139) are also slightly kinked. However, TM V in the holo protein is very stable most probably owing it to the presence of PK11195, though its binding site is distant from P139.

The root mean squared fluctuations (RMSF) of Cα atoms were calculated in the range of 400 ns to 1000 ns, when both systems reach equilibration. The RMSF for both systems are very similar, but one can note important peaks at residues A35, E70, and F100 in the apo model (Figure 4b). These regions correspond to the first three loops fluctuating more in the apo than in the holo protein.

In the *h*TSPO–PK11195 complex, LP I with the small α-helix (residues G28 to G36) in the middle of it is stable during the whole MD simulation time. A crucial role in its high stability is played by a patch of interactions (described in details in the previous section) that hinders the free movement of LP I and contributes to the α-helix retaining its conformation. In contrast, LP I in the apo model varies in length (between F25–P45 and S23–P45), and the small α-helix is rarely formed. Due to its random coil structure and the absence of PK11195, LP I is more flexible than in the holo protein. LP II in the holo model is stable during the MD simulation, while in the apo protein, one helix turn at the C–terminus of TM II unfolds (data not shown), extending the length of LP II (W68–A78), which consequently fluctuates more than in the holo protein. Here, again, PK11195 seems to play an important role in the stability of TM II (Figure 4c,d).

LP III in the holo protein consists of residues G102–L109, and despite its length, it is more stable than in the apo protein where it is composed of residues F99–N104 (Figure 6). We observed that in both proteins, the parallel cation-π interactions are formed between R103 (LP III) and W33 (LP I α-helix) for more than 75% of the simulation time. Hydrophobic interaction between W33 and F100 (TM III) further stabilize the previous interaction in the holo protein (existent for 90% of the simulation time), but much less in the apo protein (existent for 40% of the simulation time). In addition, in the holo protein, F100 binds with Y34 (LP I) for 730 ns and for about 450 ns also with PK11195. This cascade stabilizes LP I, LP III, and the whole upper, C–terminal part of the holo *h*TSPO, namely the part in the outer membrane leaflet, where the ligand binding site is present. In the apo protein, this same cascade is not stabilized by PK11195, and indeed, LP III fluctuates more (Figure 6).

Finally, LP IV and LP V remain stable without changes in both models during all MD simulations, in line with our results that TM IV and TM V are the most stable helices regardless of the ligand’s presence, and their termini do not unfold, as seen for some other helices described above (Figure 4c,d).

Taken together, the PK11195 ligand appears to reduce the fluctuations of the loops and to stabilize the overall structural fold of the TSPO protein.

### 2.4. Cholesterol Interactions with *h*TSPO

Our analyses show that the apo *h*TSPO model binds 1.5 times more cholesterol molecules than the holo one. This result is in good agreement with other studies postulating that PK11195 reduces the cholesterol binding to the TSPO [7].

We analyzed the average simulation time during which cholesterol interacts with each of the five TM helices (Table 1). In the holo protein, cholesterol interacts most often with TM I (47% of the simulation time) and TM V (48% of the simulation time), while in the apo protein, it interacts for 100% of the time with TM V and much less with other helices. Among other helices, TM II stands out, binding cholesterol for about 50% of the simulation time.

**Figure 7:**
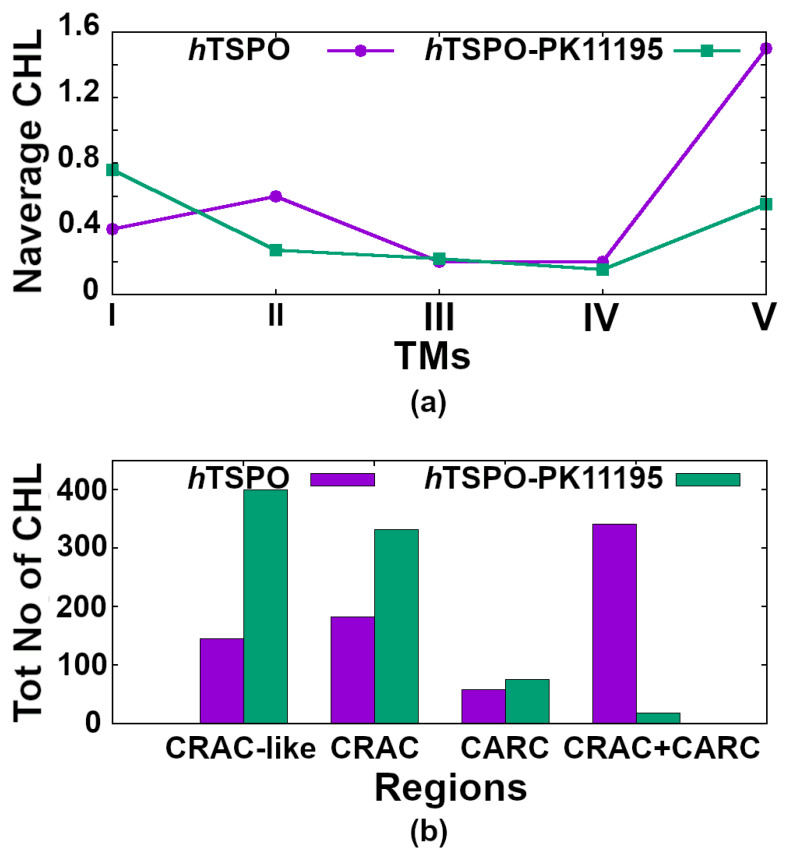
(**a**) The average number of cholesterol molecules (Naverage CHL) binding to the individual helix (TM I–TM V) in the apo (violet line) and holo (green line) *h*TSPOs at each frame of the 1 μs MD trajectory. (**b**) The total number of all cholesterol molecules (Tot No of CHL) binding either to the CRAC-like motif in TM I or to the CRAC and/or CARC in TM V during our 1 μs long MD simulation of apo *h*TSPO (violet) and holo *h*TSPO (green).

Next we calculated the average number of cholesterol molecules bound to a single TM domain per frame (i.e., per ns). The holo TM I binds on average slightly more cholesterol than TM I in the apo protein, while for TM II, the result is inverted (Figure 7a). TM III and TM IV bind on average the same amount of cholesterol per frame, but holo TM V binds less than one cholesterol per frame, while apo TM V binds on average 1.5 cholesterol molecules per frame during the full length of the simulation time (Figure 7a). Indeed, the high affinity cholesterol binding motifs, CRAC and CARC, are located in TM V (Figure 1 and Figure 2).

We analyzed why TM I of the holo protein binds cholesterol more frequently than other helices and why it binds more cholesterol than TM I in the apo protein (Figure 7b). We found that TM I has a CRAC–resembling motif, namely L17–X(2)–F20–X(3)–R24 (Figure 1), that attracts cholesterol. This motif is located in the upper, C–terminal part of the TSPO, in the outer membrane leaflet, next to the PK11195 binding site. We suggest that cholesterol binds more often to this motif in the holo than in the apo protein due to the higher stability of the TM I helix in the former protein (Figure 4c,d). The higher stability of holo TM I (i.e., it is less kinked than apo TM I (Figure 5, TM I) allows for the more optimal orientation of cholesterol binding residues L17, F20, and R24 and of cholesterol molecules with respect to the apo protein.

For TM II, the frequency of cholesterol binding is inverted: it interacts more often with the apo than with the holo protein. Cholesterol binds to both proteins only in the first half of the simulation time. Two cholesterols interact with apo TM II, one in the upper, C–terminal part (binding to residues P45–W47–V48–P51–V52) and one in the lower, N–terminal part of the protein (binding to residues T55-A59–Y62–L66). Cholesterol interacts almost twice more frequently with the lower part of the helix that is in the inner membrane leaflet than with the upper part of the helix that is in the outer membrane leaflet. In the holo protein instead, cholesterol binds exclusively to the upper part of TM II. Since apo TM II binds more cholesterol than holo, it is clear that cholesterol has higher binding affinity for the lower part of this helix (i.e., it rather binds to the N–terminal part of the *h*TSPO that is in the inner membrane leaflet than to its C–terminal part). We suggest that PK11195 reduces the frequency of cholesterol binding to the lower, N–terminal part of the *h*TSPO and in this particular case to TM II.

TM III and TM IV bind cholesterol equally frequently in both systems.

Finally, we determined the frequency of cholesterol binding to TM V, precisely to the CRAC (L150–X–Y152–X(3)–R156) and CARC (R135–X(2)–Y138–X(2)–L141) motifs. We defined how many cholesterol molecules bind individually to CRAC or CARC, as well as the frequency of cholesterol binding to both regions simultaneously (Figure 7b). For individual binding, we counted cases where only one motif at a time is occupied by cholesterol. For simultaneous binding, we counted only the cases when both motifs are occupied at the same time and with two different cholesterol molecules. Cases where one cholesterol molecule is bridging the two regions were excluded. Our results show that in the holo protein, cholesterol binds most often to CRAC and much less often to the CARC motif. Interestingly, the presence of PK11195 almost abolishes the simultaneous binding of cholesterol to both motifs.

In the apo protein, cholesterol binds more often to CRAC than to CARC. The number of cholesterol binding to these motifs is lower than in the holo protein, owing to the fact that cholesterol in the apo protein preferentially binds to both motifs at the same time.

**Figure 8:**
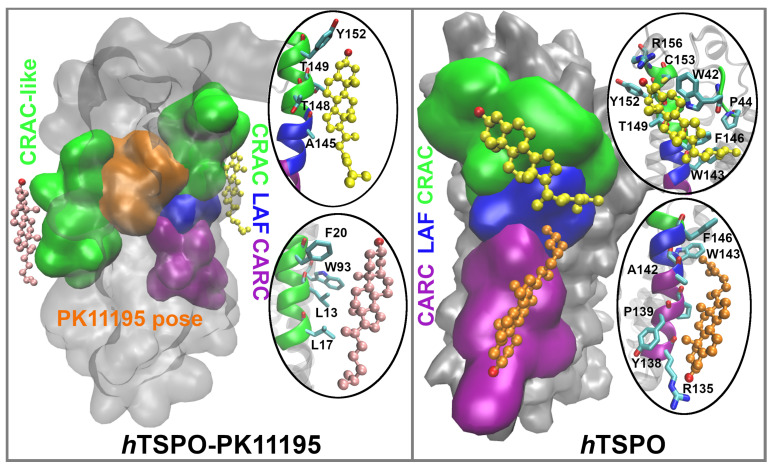
Cholesterol molecules bind most frequently to CRAC and CRAC–like regions (green surface representation) that are in the vicinity of the PK11195 binding site (orange surface representation) in the *h*TSPO–PK11195 system and to CRAC, LAF(blue surface representation), and CARC (purple surface representation) motifs in the *h*TSPO system. Corresponding residues from each region that interact with cholesterol (color coded, respectively) are represented within ellipses.

Taken together, the apo protein binds more cholesterol molecules than the holo protein. In the apo protein indeed, cholesterol binds with about 50% frequency to TM II and with 100% frequency to the CRAC and CARC regions in TM V. In TM II, cholesterol binds more readily to the lower part of the helix, so to the N–terminal part of *h*TSPO present in the inner membrane leaflet. Very interestingly, in the apo protein, cholesterol binds most frequently to CRAC and CARC simultaneously (Figure 8, right panel), while in the holo protein, simultaneous binding to these motifs is almost abolished. Indeed, cholesterol in the holo protein binds mostly to the newly described motif in TM I and to the CRAC motif in TM V (Figure 8, left panel). Both motifs are present in the upper, C–terminal part of the *h*TSPO, next to the PK11195 binding site and in the outer membrane leaflet. In our study, the binding of cholesterol to the lower, N–terminal part of the holo *h*TSPO model is rarely observed.

## 3. Materials and Methods

### 3.1. Building the 3D Structural Model of *h*TSPO

We used the sequence reported in the UniProtKB [47,48] database with the ID: P30536. It contains 169 amino acids, spanning from M1 to E169. This sequence was aligned with those of: *Mus musculus* TSPO (*Mo*TSPO; UniProt ID: P50637), *Rhodobacter sphaeroides* TSPO (*Rs*TSPO; UniProt ID: Q9RFC8), and *Bacillus cereus* TSPO (*Bc*TSPO; UniProt ID: Q81BL7), using the Multalin [70] and ClustalO [46] web-servers. 3D structural models of the *h*TSPO were built based on the *Rs*TSPO template (PDB ID: 4UC1 [35]). Twenty models of *h*TSPO were generated using the MODELLER program, Version 9.19 [71]. All models were analyzed according to the Discrete Optimized Protein Energy (DOPE) score using the built-in script of the MODELLER package [71,72]. In addition, the local structural quality of the *h*TSPO models in the biological membrane were examined using the QMEANBranescoring function [73] from the Swiss-model server [74,75,76,77,78]. All models were visually inspected and compared to the template and to the available mutagenesis data. The model corresponding best to the available experimental data, having the lowest DOPE score according to the MODELLER program [71,72] and the appropriate local structural quality as defined by the QMEANBrane tool [73], was chosen for docking and molecular dynamics (MD) simulation [79,80] studies. We checked that the orientation and tilt angles of the helices—once the model is inserted in a membrane—were appropriate. These parameters were computed by the Positioning the Proteins in Membranes (PPM) server [81] for the model and the *Rs*TSPO template and compared between them (Table 2).

### 3.2. Docking of the PK11195 Ligand

We docked the PK11195 ligand (*N*-[(2R)-butan-2-yl]-1-(2-chlorophenyl)-*N*- methylisoquinoline-3-carboxamide) to the *h*TSPO structural model. The initial 3D structure of PK11195 was obtained from the PubChem database (https://pubchem.ncbi.nlm.nih.gov/compound/1345 (accessed on 1 February 2019)). Molecular docking was performed using the UCSF Chimera program [82] and AutoDock Vina package [59]. Protein and ligand input files were prepared by AutoDockTools. The ligand had fully flexible torsion of freedom, while the receptor side chains were kept rigid. Non-polar hydrogen atoms of the protein and the ligand were merged. The center of the grid was placed at X = −14.451 Å, Y = 25.618 Å, and Z = 25.297 Å. The grid dimensions were 76 × 72 × 66 Å, and the spacing between the grid points was set to 0.375 Å. The exhaustiveness parameter of the global search was set to 8 (default). Ten ligand binding modes were generated in search for a ligand pose with the lowest binding affinity.

We selected the model where the ligand had the lowest, i.e., the most negative docking binding affinity score, and it interacted with the two conserved residues W53 and W95 [57] shown to be important for binding [34].

### 3.3. Molecular Dynamics Simulations

The apo (*h*TSPO) and holo (*h*TSPO–PK11195 complex) models were then inserted into the lipid bilayer composed of phosphatidylcholine (POPC)—phosphatidylethanolamine (POPE)—cholesterol (CHL) with the ratio of 3:3:1 for POPC:POPE:CHL, respectively. The choice of the membrane composition was made based on the experimental studies of the mitochondrial membrane and the protein–lipid monolayers [83,84]. The membrane thickness was 3.04 nm and was built by the Mem-Builder web-server [85,86]. The *h*TSPO models were placed and properly oriented at the center of the membrane box by the Lambada and InflateGRO2 tools [87] (the tilt angle of all TM helices is 10${}^{\circ }$). Principally, these values correspond to those of the *Rs*TSPO template (Table 2).

The apo and holo models of *h*TSPO inserted in the POPC–POPE–CHL membrane were solvated with 12,758 water molecules enclosed in a solvation box with dimensions of 10.5 nm × 10.5 nm × 11.0 nm. 161 sodium ($Na^{+}$) and 166 chloride ($Cl^{-}$) ions were added to neutralize the system net charge and to reproduce the physiologic electronic strength of 0.15M. The MD simulations were run using the GROMACS 2018.6 package [88,89] and applying the SLIPIDSforce field [90] for the membrane, the AMBER99SB-ILDNforce field [91] for the *h*TSPO model and ions, and the TIP3P [92] force field for water. The force field parameters of PK11195 were prepared using the General Amber force field (GAFF) [93,94], introducing the RESPatomic charges and electrostatic potential (ESP) as calculated based on the B3LYP/6-31G* basis set using the Gaussian09 package [95]. The topology file of the PK11195 ligand was converted to GROMACS format using the ACPYPE tool [96]. The geometry of the *h*TSPO models was optimized by steepest descent minimization performed for 50,000 steps with a maximum force constant value of 1000 kJ/mol/nm. After the geometrical optimization, the systems underwent NPTequilibration for 10 ns with a time step of 2 fs. The systems were maintained at the reference pressure of 1 bar by coupling to the Parrinello–Rahman barostat [97,98] with uniform scaling of x-y box vectors and independent scaling for the z-axis (i.e., perpendicular to the membrane). The systems were coupled to the Nose–Hoover thermostat [99,100,101] to maintain the temperature at 310 K. A 1.2 nm cut-off was set for the short-range non-bonded interaction. The LINCSalgorithm [102] was chosen to constrain all bonds involving hydrogen atoms. The holo and apo structural models of *h*TSPO were then simulated for 1 μs to detect the stable structure of *h*TSPO in the mitochondrial membrane. MD simulation parameters were the same as in the NPT equilibration run; only the thermostat was changed to the V-rescale thermostat [101].

### 3.4. Analysis

**The root mean squared deviation (RMSD)** for the entire *h*TSPO model and for the individual helices was calculated for the backbone atoms omitting the hydrogen atoms. The RMSD for the entire protein was calculated for the sequence from W5 to N158, excluding the N– and C–termini and the hydrogen atoms.

**The root mean squared fluctuation (RMSF)** was calculated for the Cα atoms of each *h*TSPO residue. They were calculated for equilibrated proteins, that is in the MD simulation range 400 ns–1 μs. For these calculations, the *g_rms* and *g_rmsf* tools from the GROMACS package were used [88,89].

**Helices flexibility analyses** was done by the Bendix plugin [67] in the VMD program [50]. To define the color code, we saved the values of the angle changes along each helix during the MD simulation time. The average values ranged from 0${}^{\circ }$ to 24${}^{\circ }$; therefore, we divided the color code into 5 parts with the corresponding 6 angle values’ extents. The highest change in the helical angle was observed for the TM I helix in the apo *h*TSPO model, i.e., 51${}^{\circ }$. The color code is described as blue: <6${}^{\circ }$, cyan: 6–12${}^{\circ }$, green: 12–18${}^{\circ }$, yellow: 18–24${}^{\circ }$, and red: >24${}^{\circ }$.

**The principal component analysis (PCA)** was performed on the apo and holo *h*TSPO including residues W5 to N158. The C– and N–termini, as well as hydrogen atoms were ignored. We used the *g_covar* and *g_anaeig* tools in the GROMACS package [88,89].

**The analysis of PK11195 interactions** with the *h*TSPO model (Figure 3) was carried out by home-made TCLand AWKscripts. To define a binding site of the ligand, we searched for all residues that were within 4.5 Å of any PK11195 heavy atom. We defined residues that bind PK11195 ligand for more than 90% of the simulation time as constant or principal binders. All of them form hydrophobic or stacking interactions with the ligand. For W53 that is H-bonding the carbonyl oxygen of PK11195, we calculated the frequency of H-bond formation using the distance criteria of 3.5 Å. The residues binding the PK11195 ligand for more than 90% of the simulation time are shown in the VMD representation in Figure 3. Residues that interact with the PK11195 for at least 75% were determined as frequent binders. All residues binding PK11195 for at least 75% are shown in the 2D plots in Figure 3. The subfigures in Figure 3 were made using the Visual Molecular Dynamics (VMD) [50] and Discovery Studio Visualizer [62] programs.

**The cholesterol analysis** was done by in-house written TCL and AWK scripts. The cholesterol was counted as bound to the *h*TSPO model, to the defined TM helix, or to different motifs (CRAC-like in TM I, CRAC/CARC in TM V) if it was found within 5 Å from any residue belonging to the analyzed region, respectively. Only contacts between heavy atoms were taken into account.

The total number of cholesterols bound to the apo and holo *h*TSPO systems throughout the 1 μs were counted and expressed as the ratio of the cholesterol molecules interacting with each system.

The data in Table 1 were obtained by counting all frames where the cholesterol interacts with the transmembrane helix in question. The percentage of the simulation time was calculated as the number of frames divided by the total number of frames (1000).

To obtain the average number of cholesterol molecules that bind the individual helix at each frame (as reported in Figure 7a), we counted the total number of cholesterol molecules interacting with the individual TM helix, and we divided this number by the total number of frames (1000).

For Figure 7b, we just counted the total number of cholesterol molecules that bind only to the CRAC–like motif in TM I, only to CRAC/CARC in TM V, or to both motifs (CRAC+CARC in TM V) at the same time.

## 4. Conclusions

The interplay between PK11195 and cholesterol interactions with *h*TSPO were studied by MD simulations of a homology model of the protein based on *Rs*TSPO. The ligand increases the stability of the protein in terms of RMSD, PCA, and Bendix analyses. During the MD simulation, PK11195 slides to a new position, in which its CL-phenyl ring initially facing TM I is placed between TM I and TM II, closer to the latter helix (Figure 3 and Figure S2).The two helices detach from one other, while they stay close to each other in the apo protein. The ligand forms mostly hydrophobic and stacking interactions with the protein. Its carbonyl oxygen forms an H-bond with the W53 side chain. Two and three cholesterol molecules per ns bind, on average, to the holo and apo *h*TSPO, respectively. Hence, the presence of the ligand reduces the frequency of cholesterol binding to the protein. In the apo protein, the cholesterol molecules bind most of the time simultaneously to two well-known cholesterol binding motifs, CRAC and CARC in TM V. In the holo protein instead, cholesterol interacts with the CRAC–like motif in TM I and with the CRAC motif in TM V. Cholesterol binds much more rarely to the lower, N–terminal part of the holo *h*TSPO, that is in the inner membrane leaflet. Thus, PK11195 reduces cholesterol binding to this latter region, but it favors cholesterol interactions with the upper, C–terminal part of the protein in the outer membrane leaflet. Further studies are required to understand more in detail why this is the case.

## Figures and Tables

**Table 1:** The percentage (%) of the simulation time during which the individual transmembrane helices (TM I–TM V) bind the cholesterol molecule(s). The total simulation time is 1 μs.

	TM I	TM II	TM III	TM IV	TM V
**Apo system**	32	51	23	27	100
**Holo system**	47	26	19	15	48

**Table 2:** Comparison of the hydrophobic thickness and protein tilt angles for the *Rs*TSPO template and the *h*TSPO structural model. All values were obtained from the Positioning the Proteins in Membranes (PPM) server [81].

Model/Template	Hydrophobic Thickness	ΔG Transfer	Tilt Angle
	(Å)	(kcal/mol)	(∘)
*h*TSPO	30.4 ± 4.1	−23.9	10.0 ± 1.0
*Rs*TSPO	30.2 ± 1.6	−40.8	7.0 ± 3.0

## Data Availability

MD trajectories and protein models are available upon request to the corresponding authors.

## Supplementary Materials

The following data are available online: Table S1: Experimentally defined dissociation constants ($K_{d}$) of the PK11195 molecule for different TSPOs. Figure S1: 3D and 2D representations of the PK11195 binding poses obtained by docking. Figure S2: MD snapshots showing the PK11195 ligand in its binding pocket at 0 ns, 250 ns, 500 ns, 750 ns and 1000 ns. Figure S3: Apo hTSPO does not exhibit an open access channel to the ligand binding pocket (located between the TM I and TM II helices in the holo protein). Figure S4: The number of cholesterol molecules binding to each helix of the apo and holo *h*TSPO proteins, averaged over our three MD simulations. Figure S5: The number of cholesterol molecules binding to apo and holo *h*TSPO averaged over our MD trajectories. Figure S6: Ligands motion in the binding site in our three MD simulations. Figure S7: Open access channels to the ligand binding pocket in our three MD trajectories.

## Abbreviations

The following abbreviations are used in this manuscript:

TSPO	translocator protein
TM	transmembrane helix
LP	loop
PK11195	1-(2-chlorophenyl)-*N*-methyl-*N*-(1-methylpropyl)-3-isoquinolinecarboxamide
	molecule
*h*TSPO	the human TSPO
*h*TSPO-PK11195	the *h*TSPO-PK11195 complex
POPC	phosphatidylcholine lipid
POPE	phosphatidylethanolamine lipid
CHL	cholesterol
RMSD	root mean squared deviation
RMSF	root mean squared fluctuation
VMD	Visual Molecular Dynamics
MD	molecular dynamics
CRAC	cholesterol recognition/interaction amino acid consensus motif
	(L150–X–Y152–X(3)–R156)
CARC	reverse region of CRAC (R135–X(2)–Y138–X(2)–L141)
