# Supplementary figures and images for: The Interplay of Cholesterol and Ligand Binding in hTSPO from Classical Molecular Dynamics Simulations
Source: Molecules. 2021 Feb 26;26(5):1250. doi: 10.3390/molecules26051250 (PMC7956637; doi:10.3390/molecules26051250)

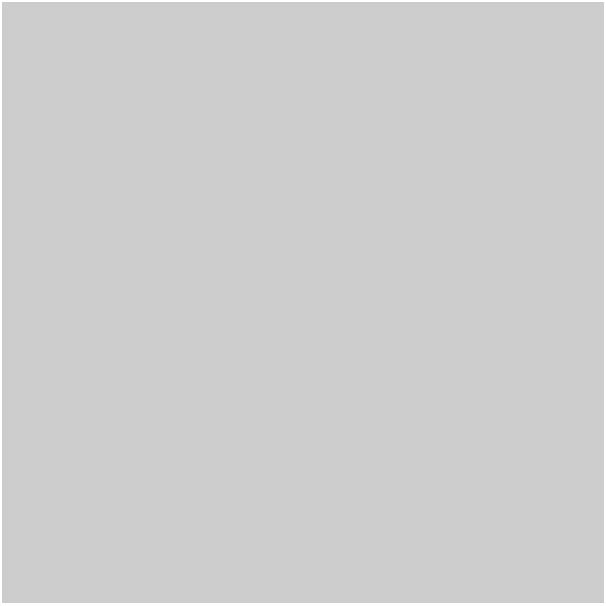

Supplement: Supplementary file 1 [file molecules-26-01250-s001.zip › molecules-1054023-supplementary/Definitions/author2.jpg]

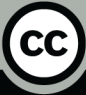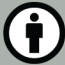

BY

Supplement: Supplementary file 1 [file molecules-26-01250-s001.zip › molecules-1054023-supplementary/Definitions/logo-ccby-eps-converted-to.pdf]

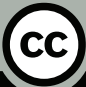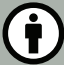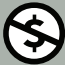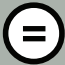

BY

NC

ND

Supplement: Supplementary file 1 [file molecules-26-01250-s001.zip › molecules-1054023-supplementary/Definitions/logo-ccby-nc-nd-eps-converted-to.pdf]

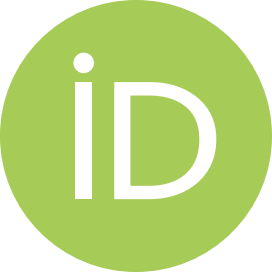

Supplement: Supplementary file 1 [file molecules-26-01250-s001.zip › molecules-1054023-supplementary/Definitions/logo-orcid-eps-converted-to.pdf]

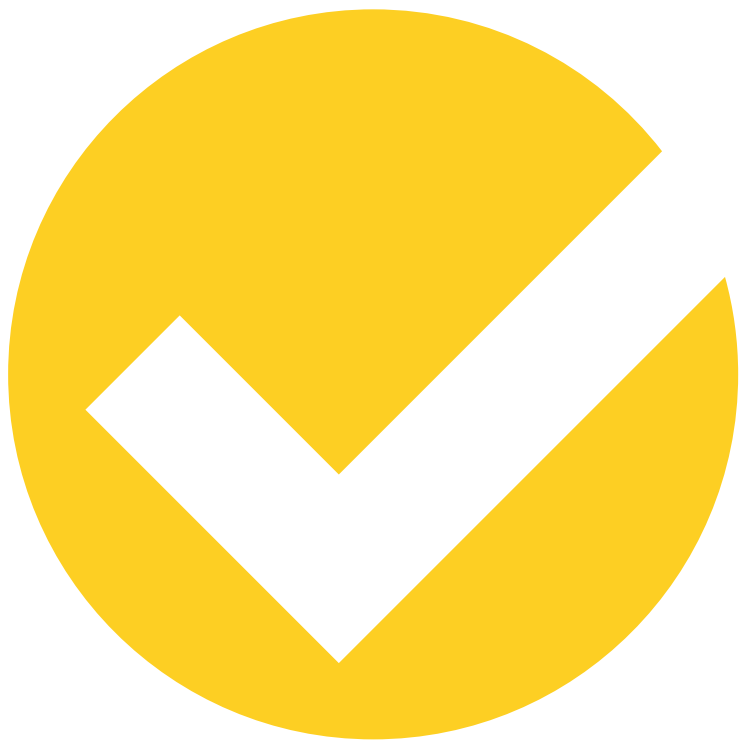

check for  
updates

Supplement: Supplementary file 1 [file molecules-26-01250-s001.zip › molecules-1054023-supplementary/Definitions/logo-updates.pdf]

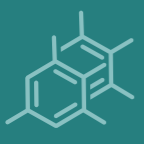

*molecules*

Supplement: Supplementary file 1 [file molecules-26-01250-s001.zip › molecules-1054023-supplementary/Definitions/molecules-logo-eps-converted-to.pdf]

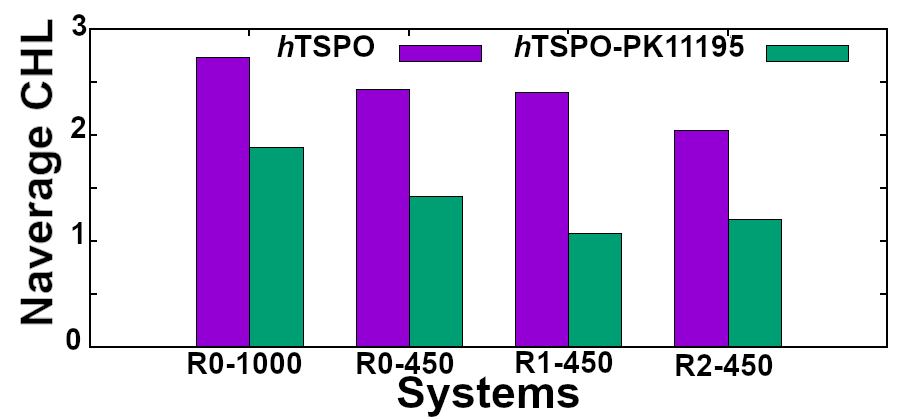

Supplement: Supplementary file 1 [file molecules-26-01250-s001.zip › molecules-1054023-supplementary/figures/4mds_aver_chl_binding_jan28.png]

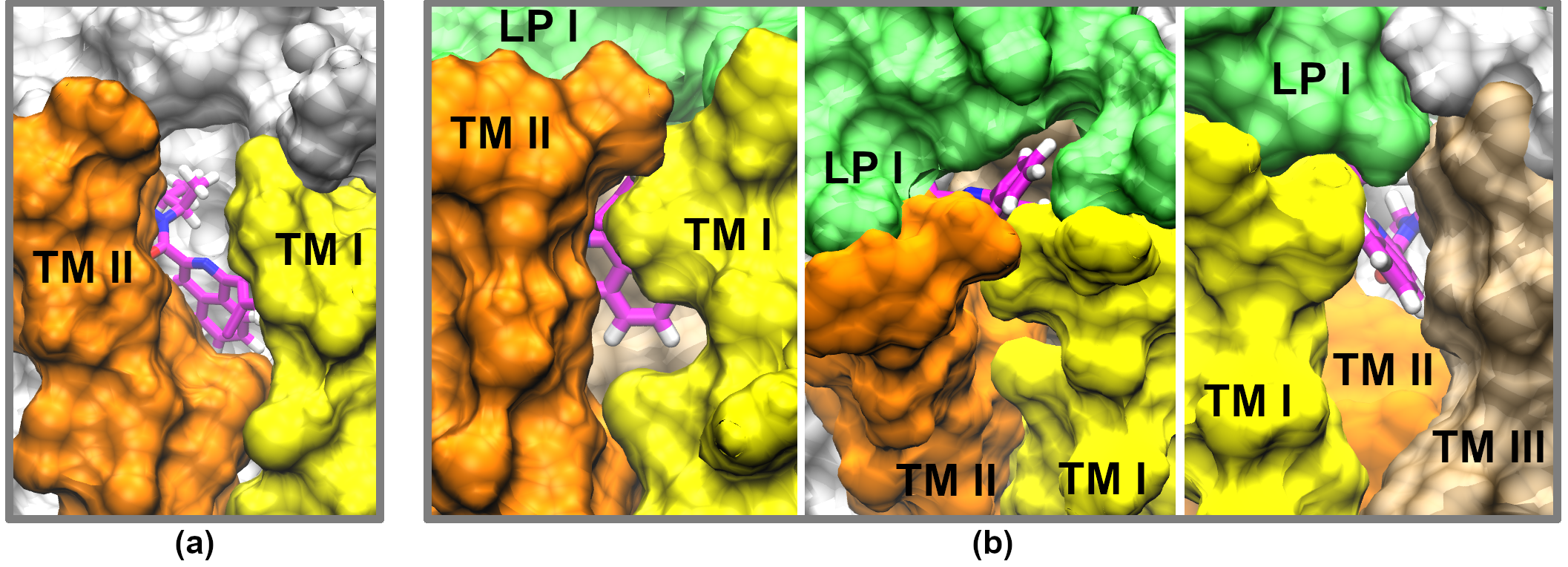

Supplement: Supplementary file 1 [file molecules-26-01250-s001.zip › molecules-1054023-supplementary/figures/4mds_opening.png]

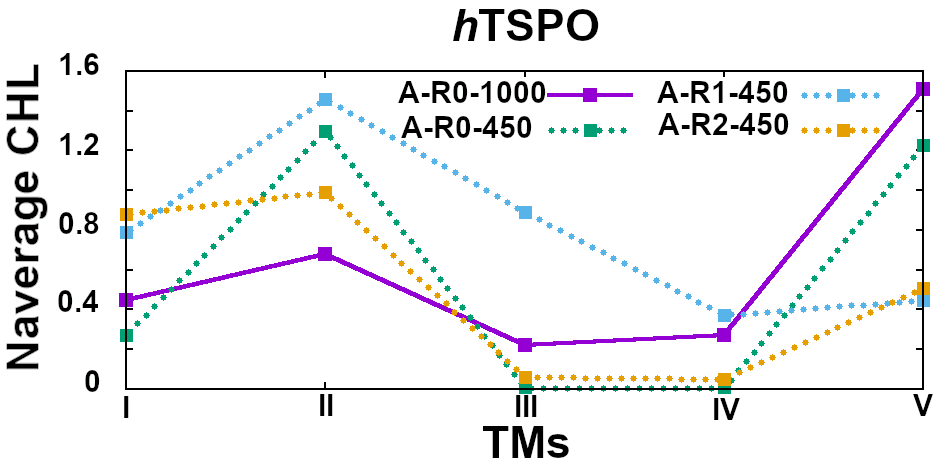

Supplement: Supplementary file 1 [file molecules-26-01250-s001.zip › molecules-1054023-supplementary/figures/4mds_plot_chl_apo_jan28.png]

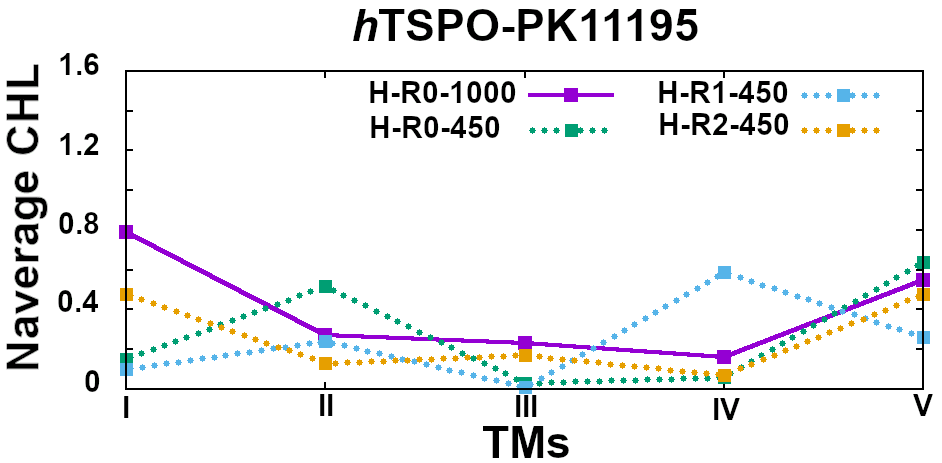

Supplement: Supplementary file 1 [file molecules-26-01250-s001.zip › molecules-1054023-supplementary/figures/4mds_plot_chl_holo_jan28.png]

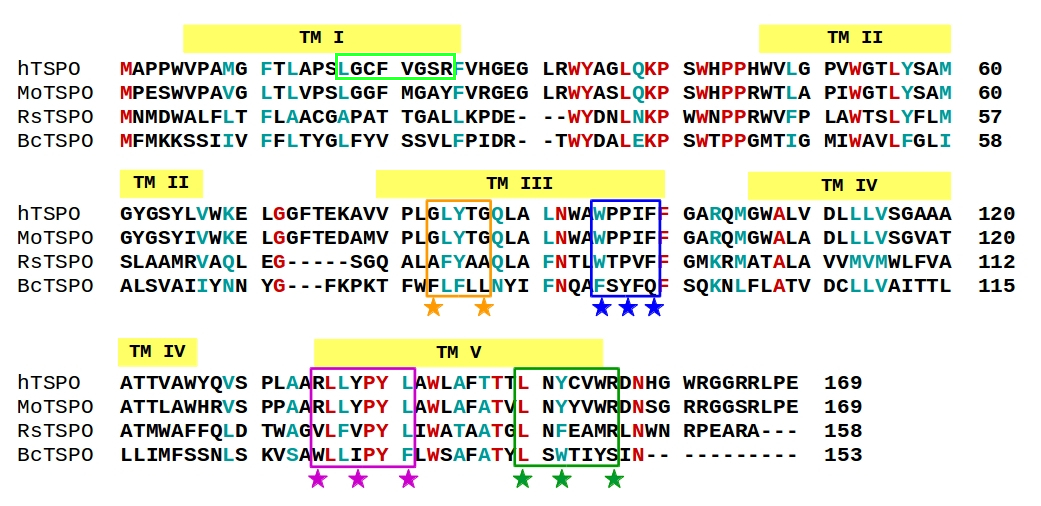

Supplement: Supplementary file 1 [file molecules-26-01250-s001.zip › molecules-1054023-supplementary/figures/align_HL_nov26.jpg]

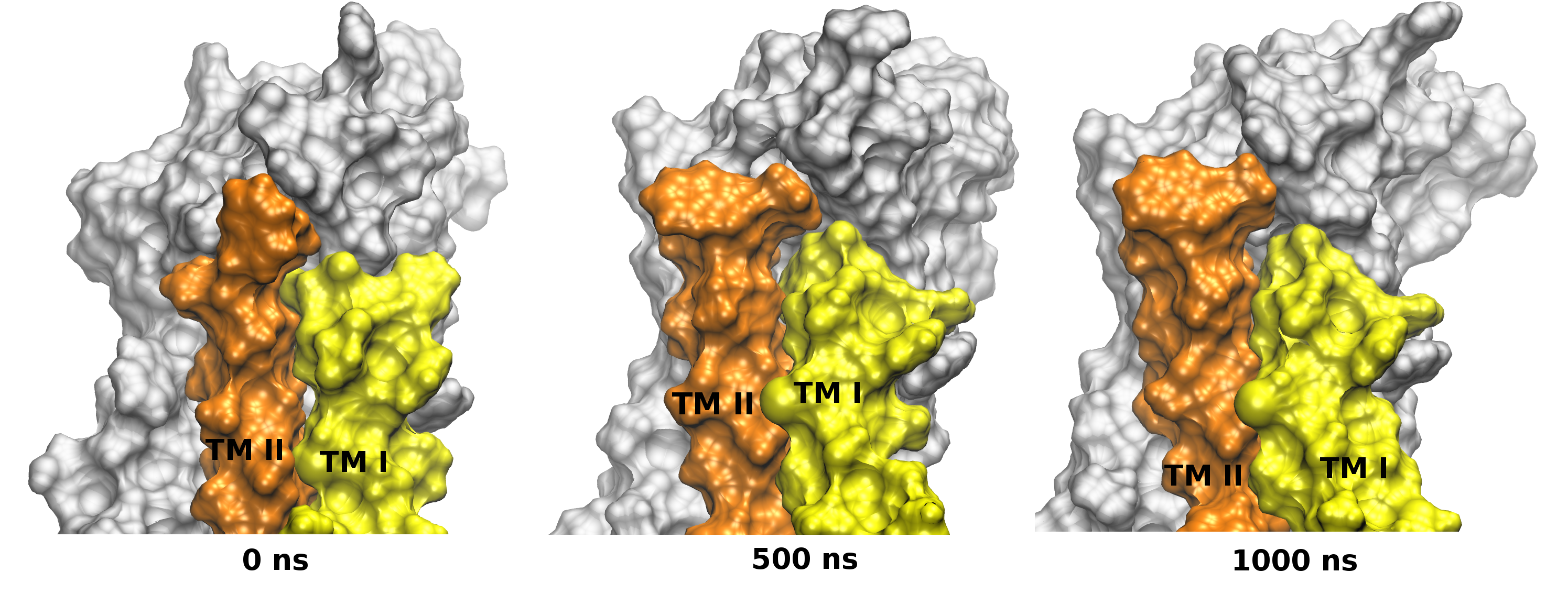

Supplement: Supplementary file 1 [file molecules-26-01250-s001.zip › molecules-1054023-supplementary/figures/apo_hTSPO_TM1-TM2_closed_conf.png]

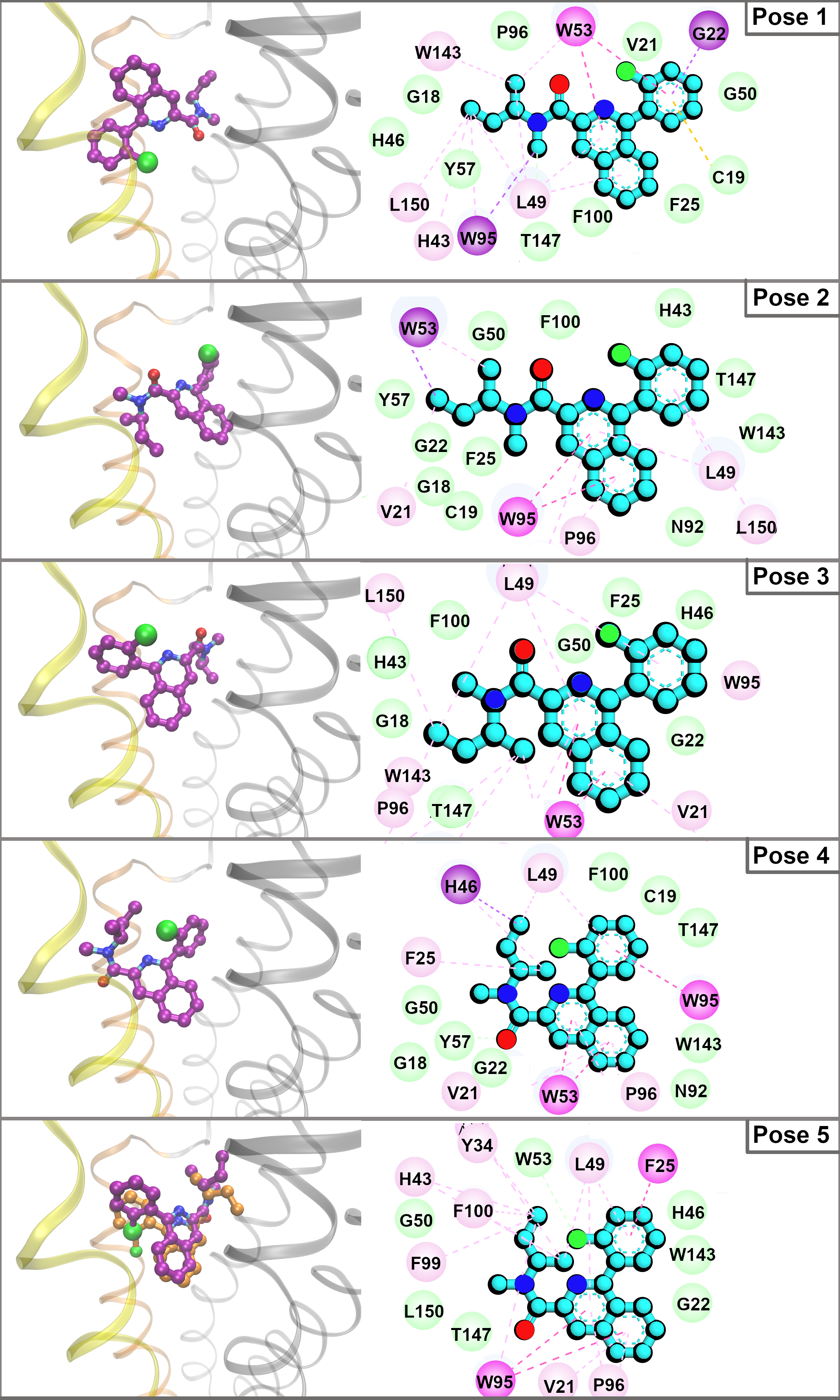

Supplement: Supplementary file 1 [file molecules-26-01250-s001.zip › molecules-1054023-supplementary/figures/docking1_jan21.png]

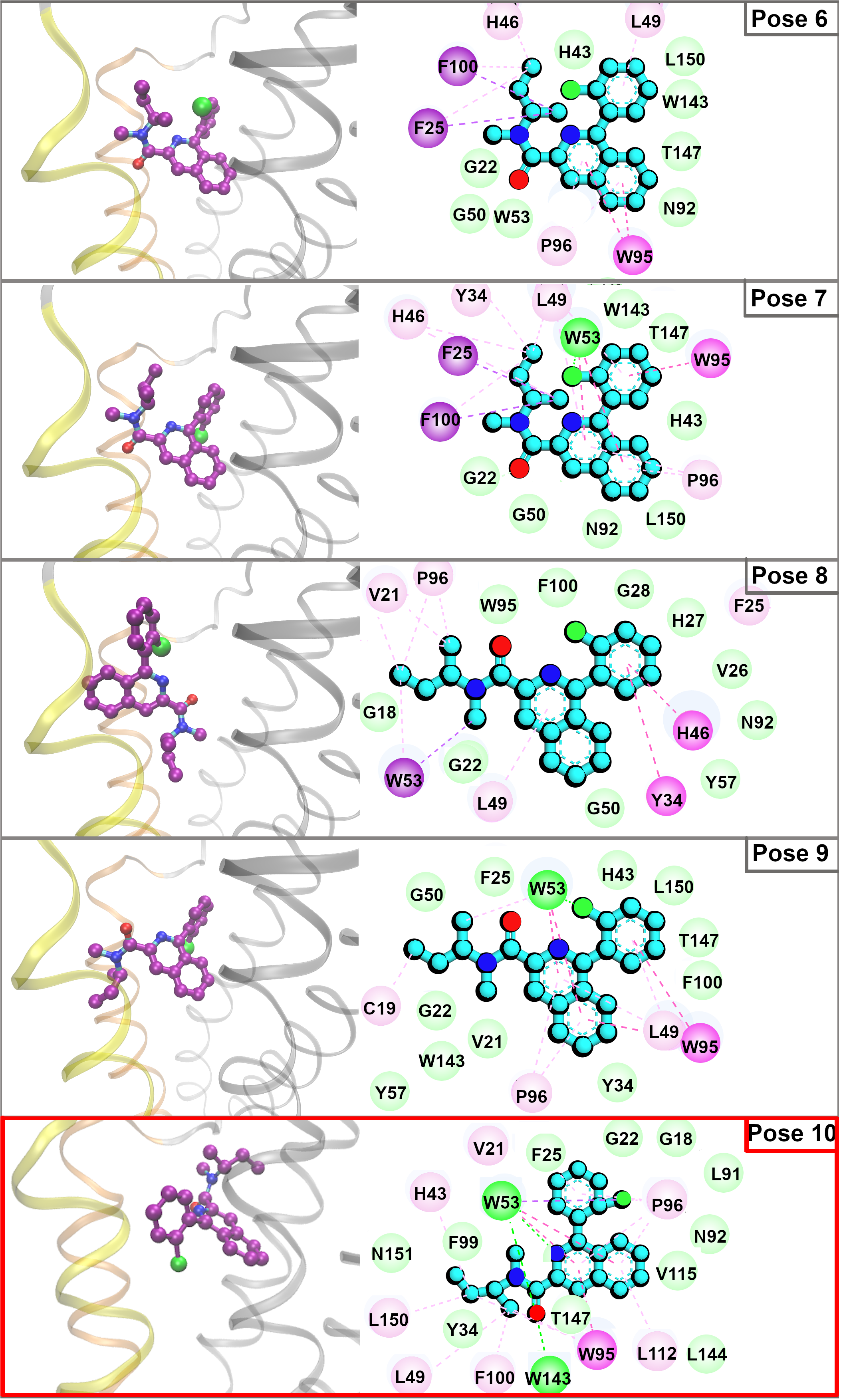

Supplement: Supplementary file 1 [file molecules-26-01250-s001.zip › molecules-1054023-supplementary/figures/docking2_jan21.png]

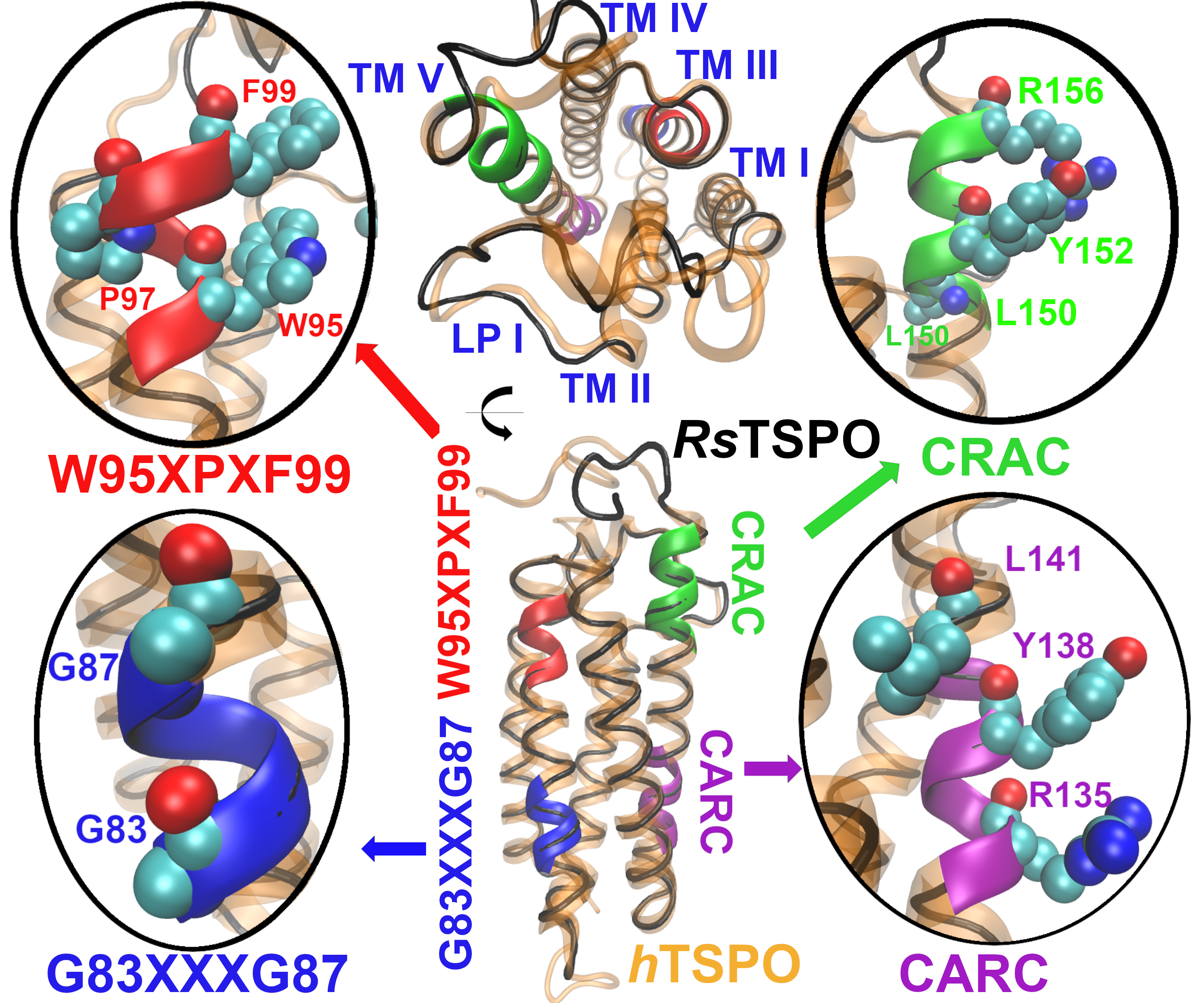

Supplement: Supplementary file 1 [file molecules-26-01250-s001.zip › molecules-1054023-supplementary/figures/figure2_dec31.png]

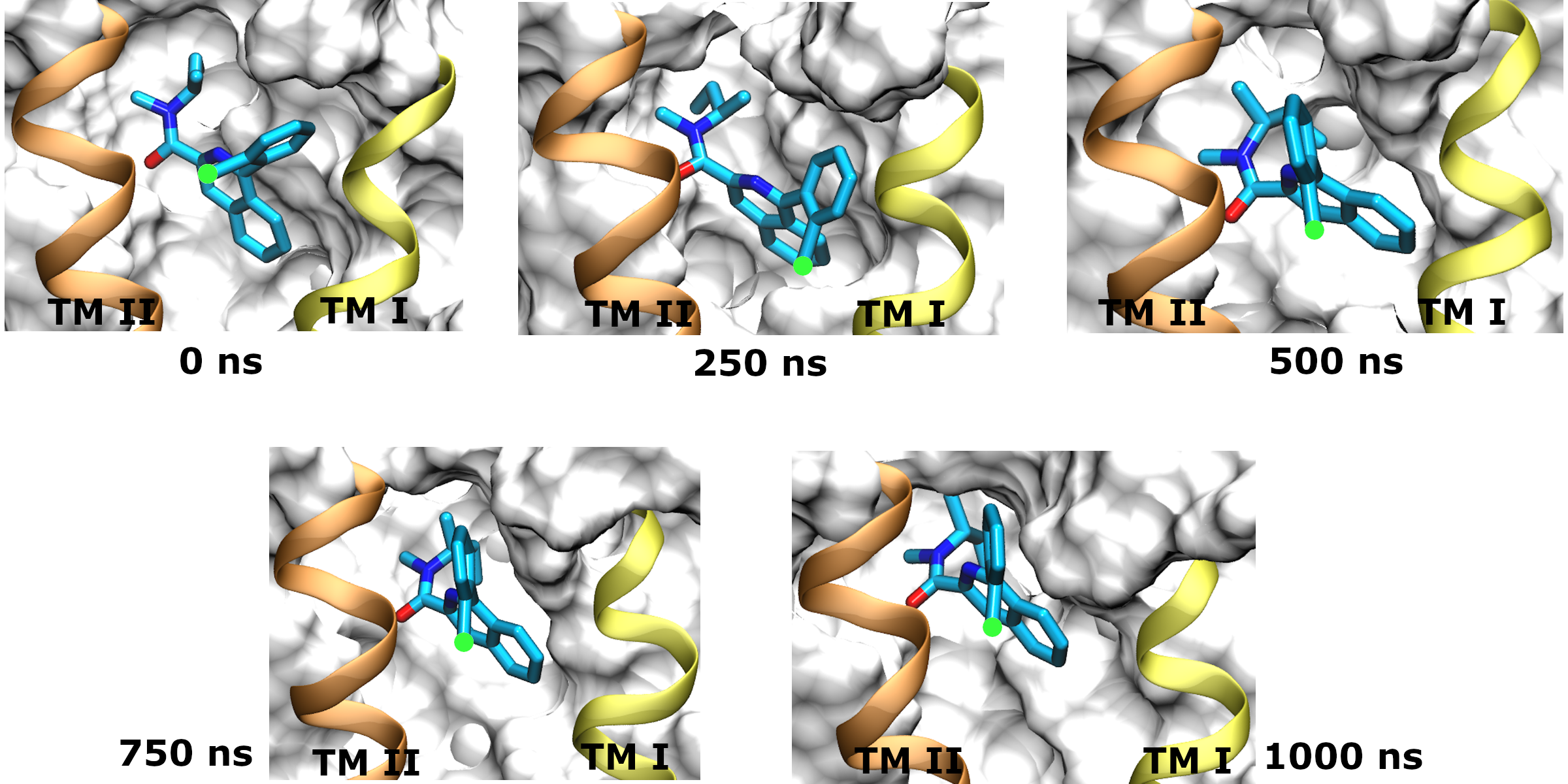

Supplement: Supplementary file 1 [file molecules-26-01250-s001.zip › molecules-1054023-supplementary/figures/Fivesnapshots-PKAinBS-16x8cm_jan28.png]

$0^{\circ}$   $6^{\circ}$   $12^{\circ}$   $18^{\circ}$   $24^{\circ}$

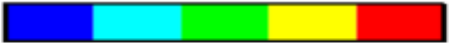

Supplement: Supplementary file 1 [file molecules-26-01250-s001.zip › molecules-1054023-supplementary/figures/legend.pdf]

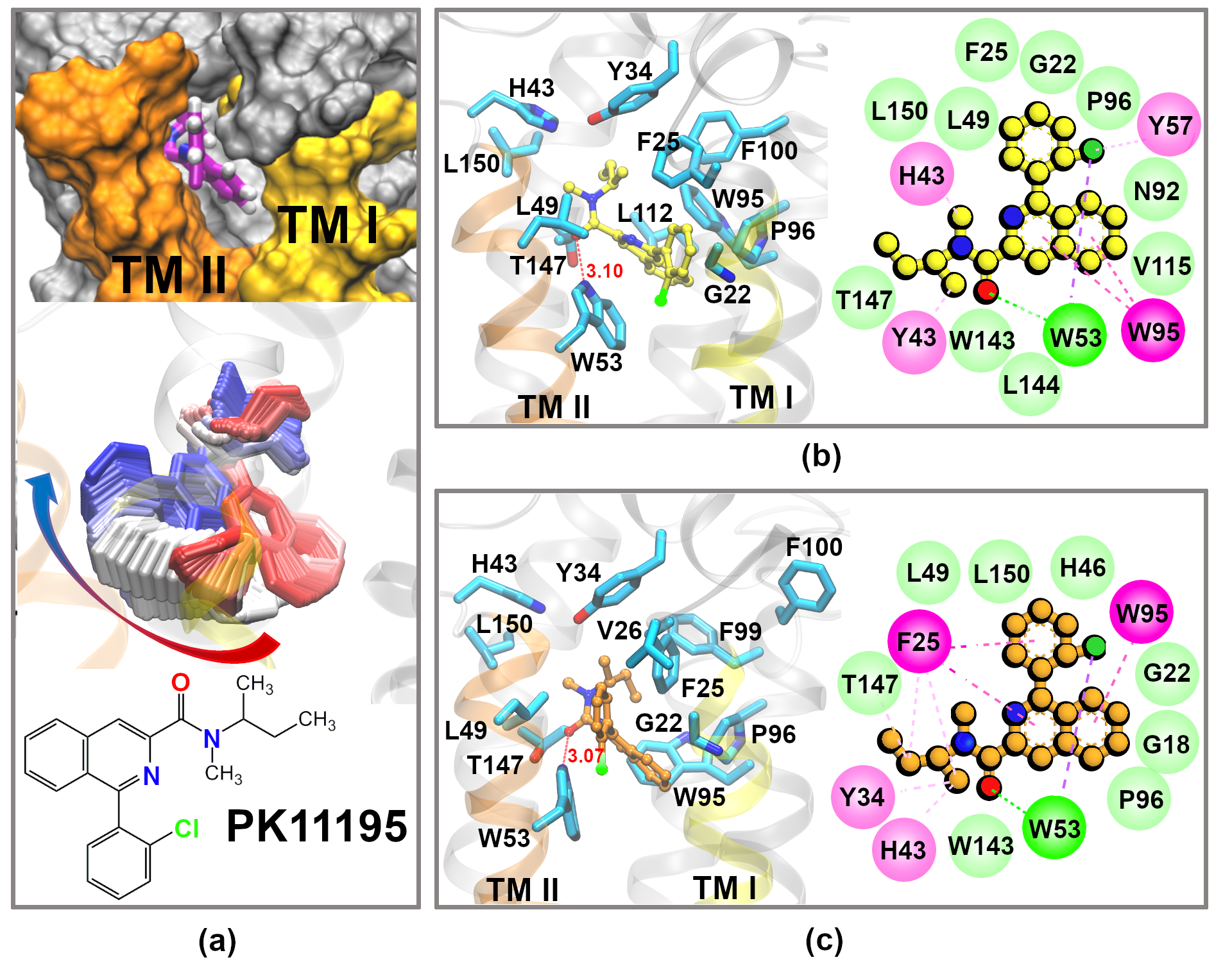

Supplement: Supplementary file 1 [file molecules-26-01250-s001.zip › molecules-1054023-supplementary/figures/make_pka_dec31.png]

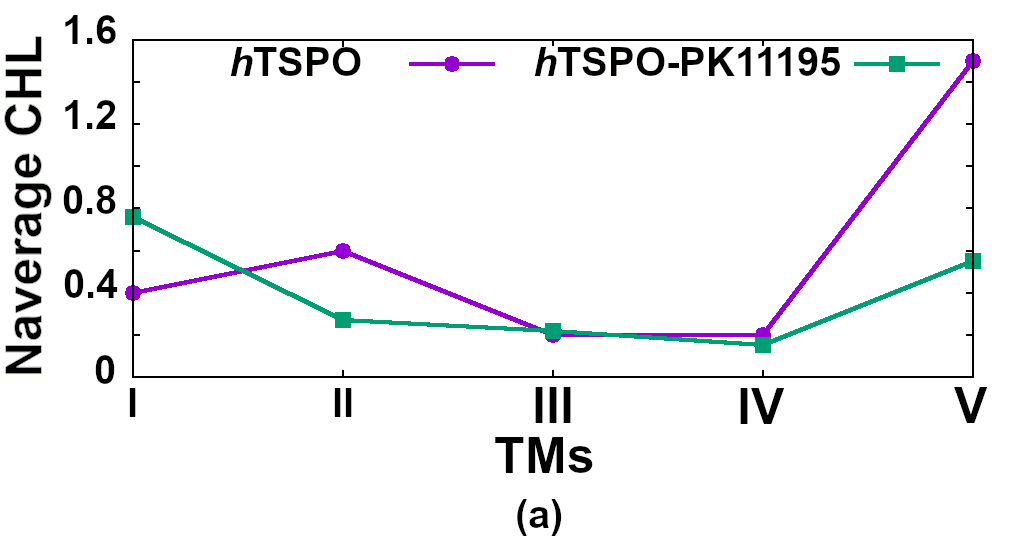

Supplement: Supplementary file 1 [file molecules-26-01250-s001.zip › molecules-1054023-supplementary/figures/nave_chl_dec31.png]

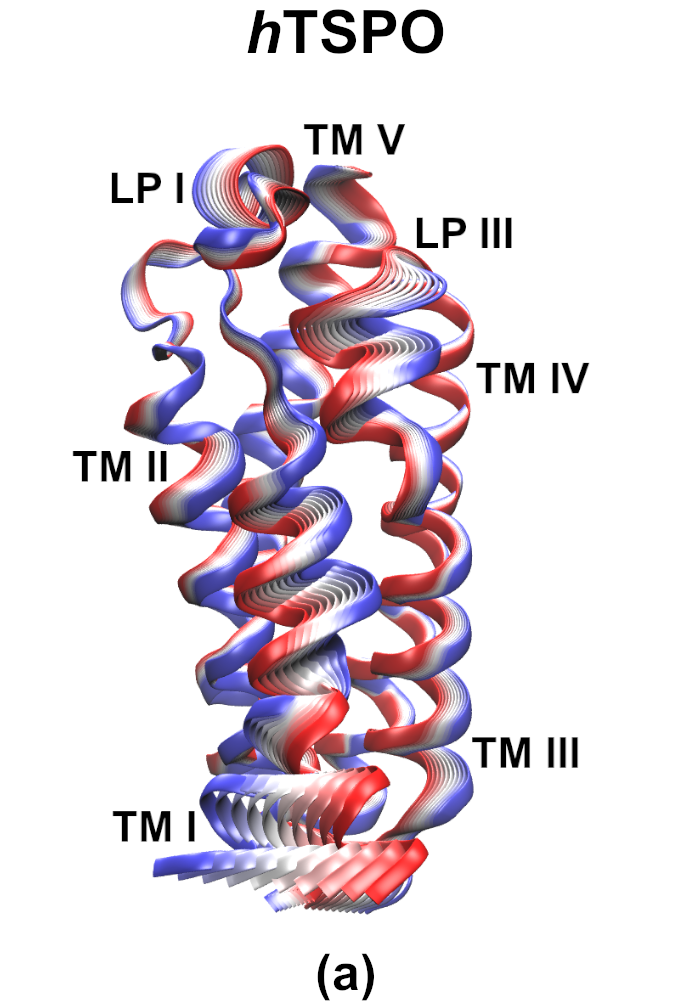

Supplement: Supplementary file 1 [file molecules-26-01250-s001.zip › molecules-1054023-supplementary/figures/pca-apo_dec31.png]

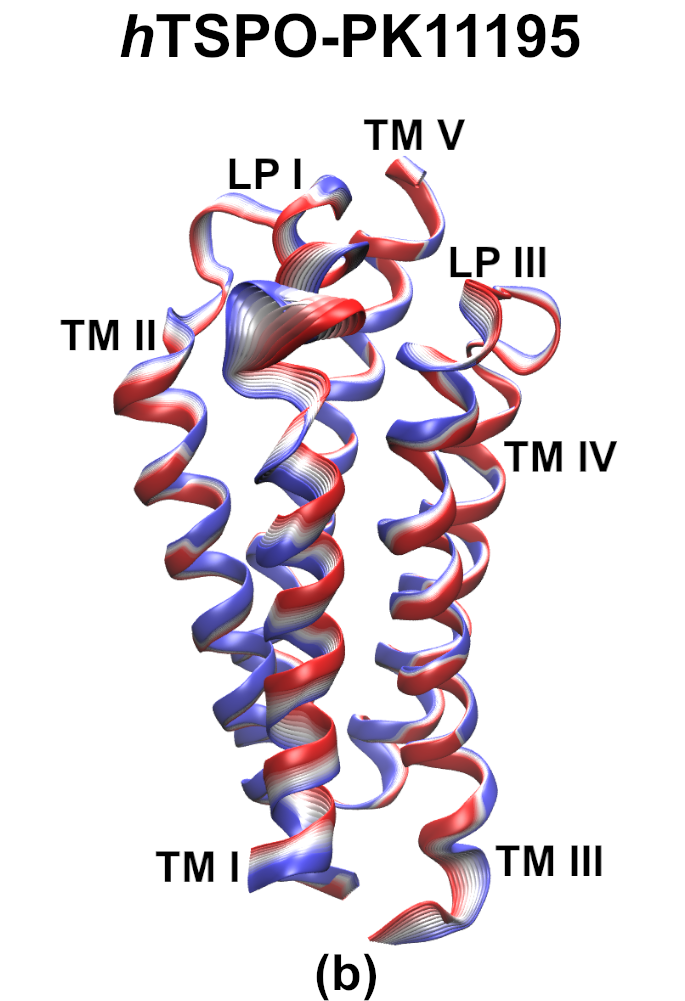

Supplement: Supplementary file 1 [file molecules-26-01250-s001.zip › molecules-1054023-supplementary/figures/pca-holo_dec31.png]

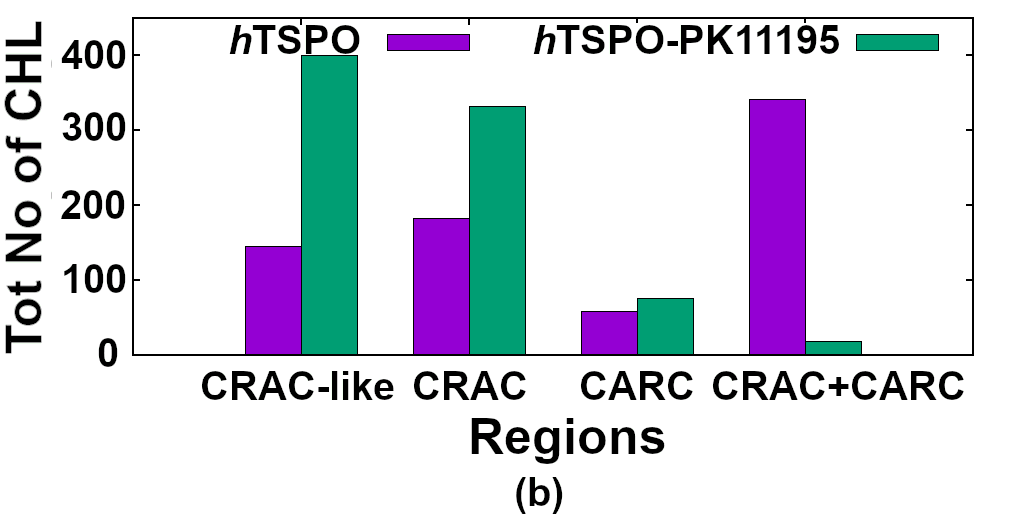

Supplement: Supplementary file 1 [file molecules-26-01250-s001.zip › molecules-1054023-supplementary/figures/plot_chl_dec31.png]

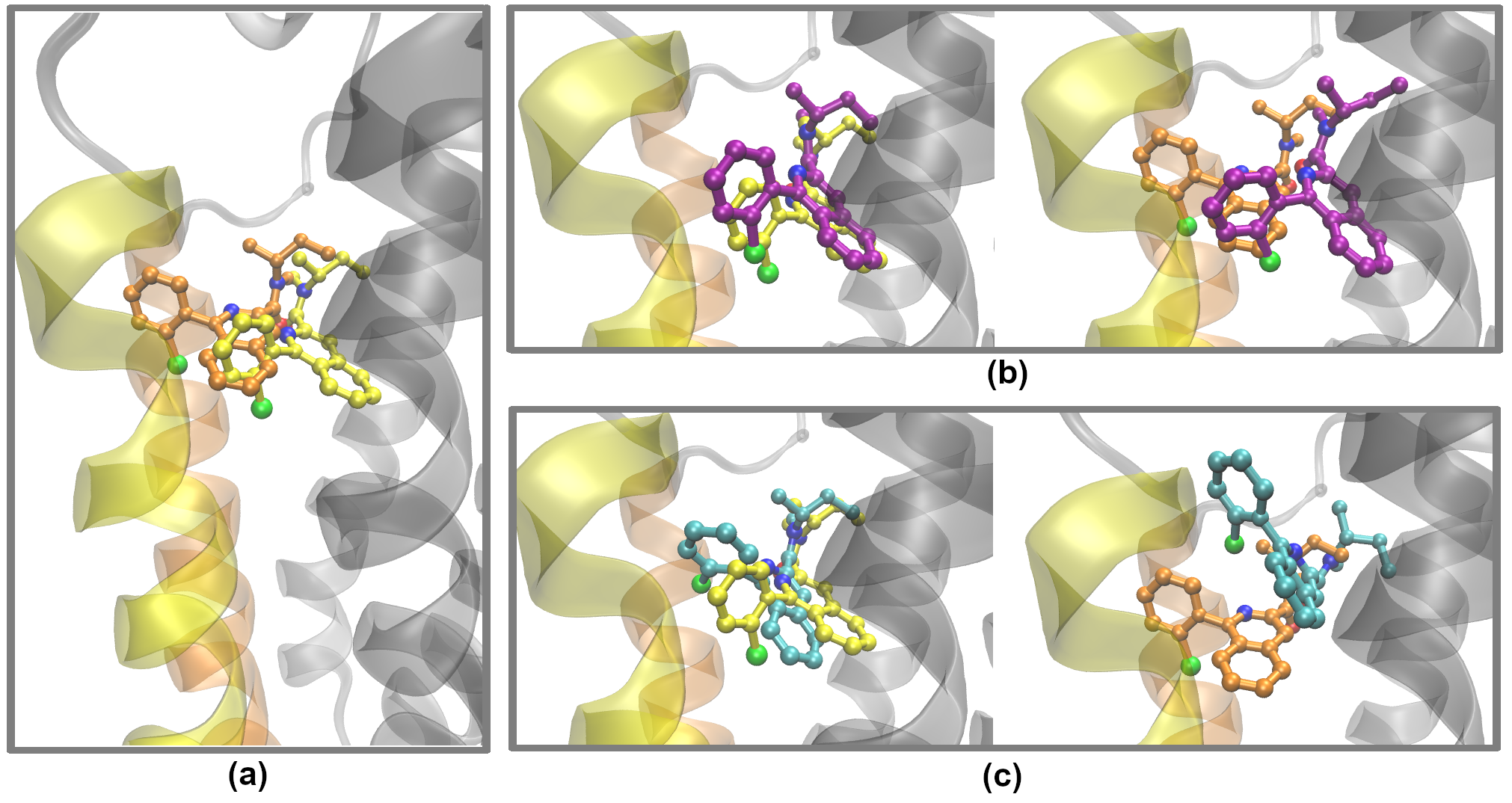

Supplement: Supplementary file 1 [file molecules-26-01250-s001.zip › molecules-1054023-supplementary/figures/replicates.png]

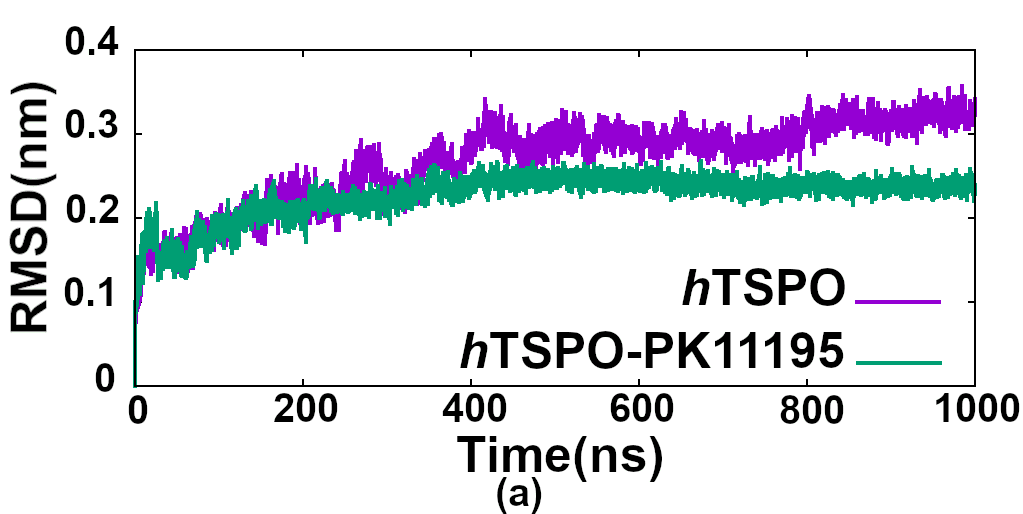

Supplement: Supplementary file 1 [file molecules-26-01250-s001.zip › molecules-1054023-supplementary/figures/rmsd_dec31.png]

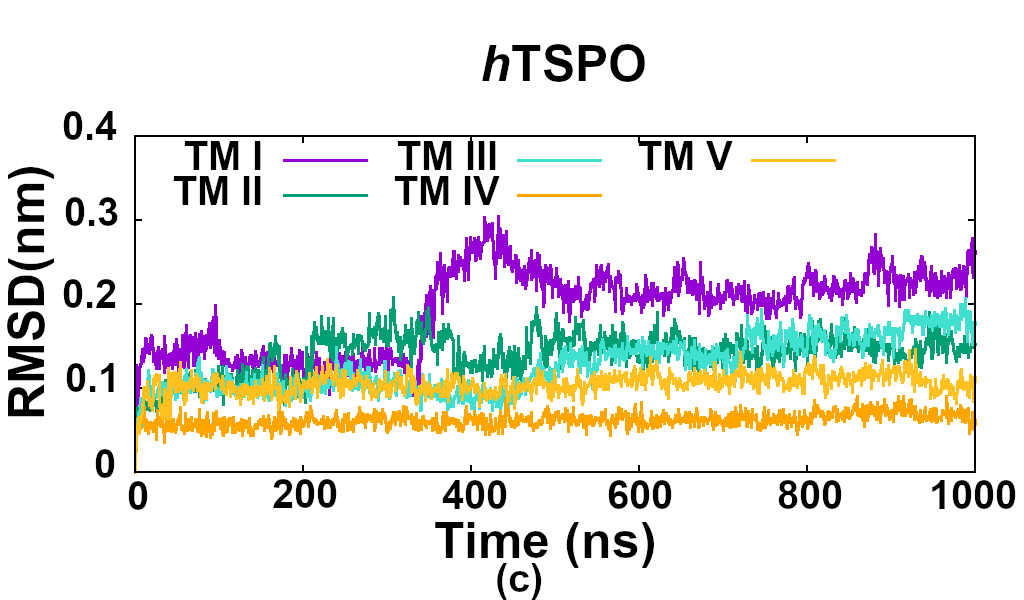

Supplement: Supplementary file 1 [file molecules-26-01250-s001.zip › molecules-1054023-supplementary/figures/rmsd_tms_dec31.png]

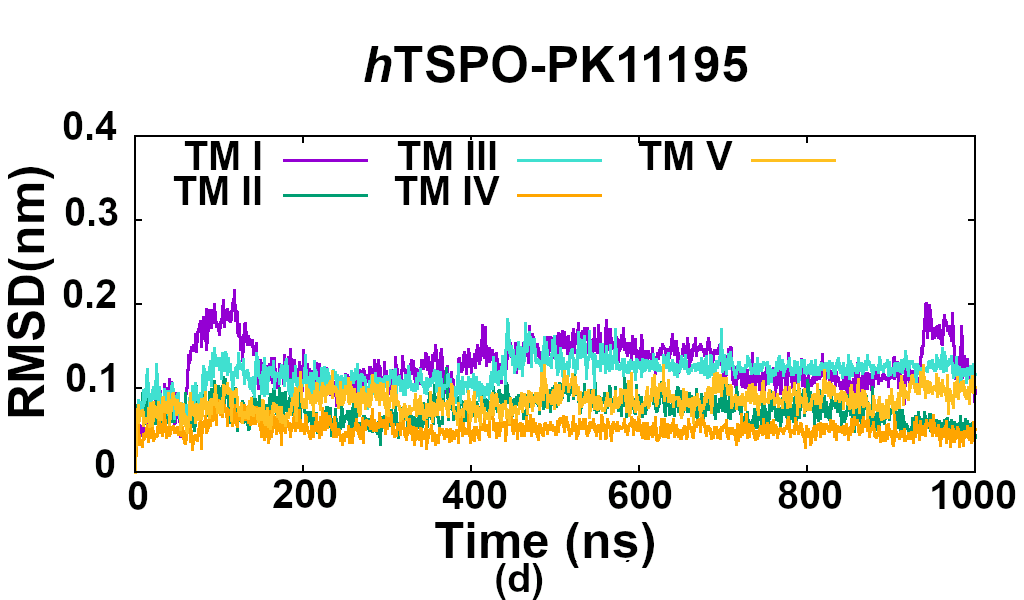

Supplement: Supplementary file 1 [file molecules-26-01250-s001.zip › molecules-1054023-supplementary/figures/rmsd_tms_PKA_dec31.png]

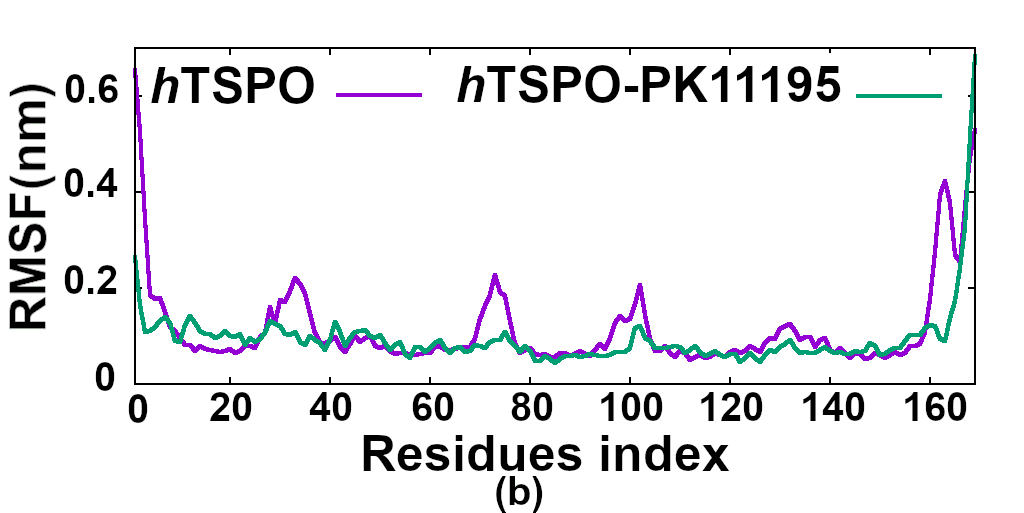

Supplement: Supplementary file 1 [file molecules-26-01250-s001.zip › molecules-1054023-supplementary/figures/rmsf_dec31.png]

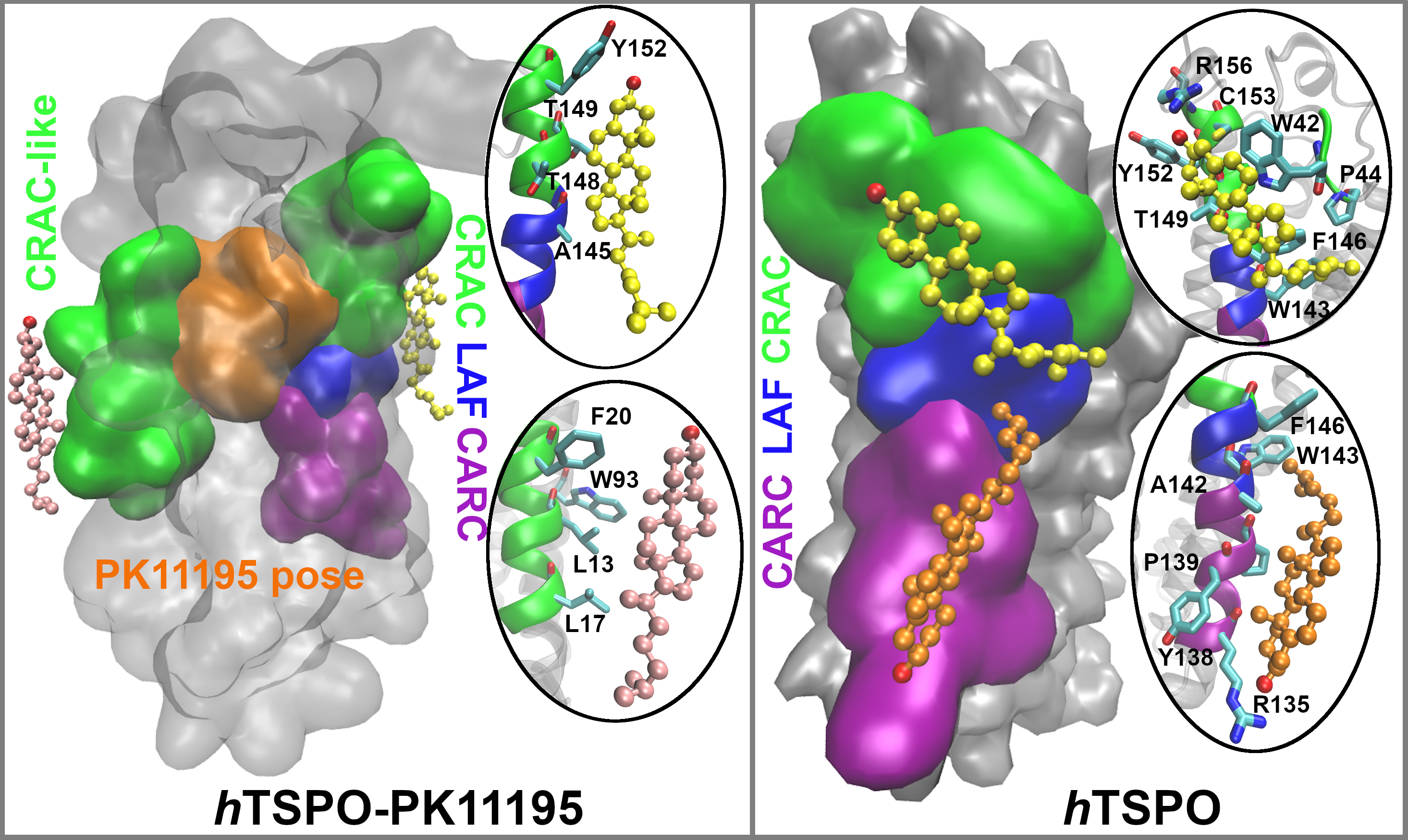

Supplement: Supplementary file 1 [file molecules-26-01250-s001.zip › molecules-1054023-supplementary/figures/sm_chl_binding_jan15.png]

# *h*TSPO

Residue index

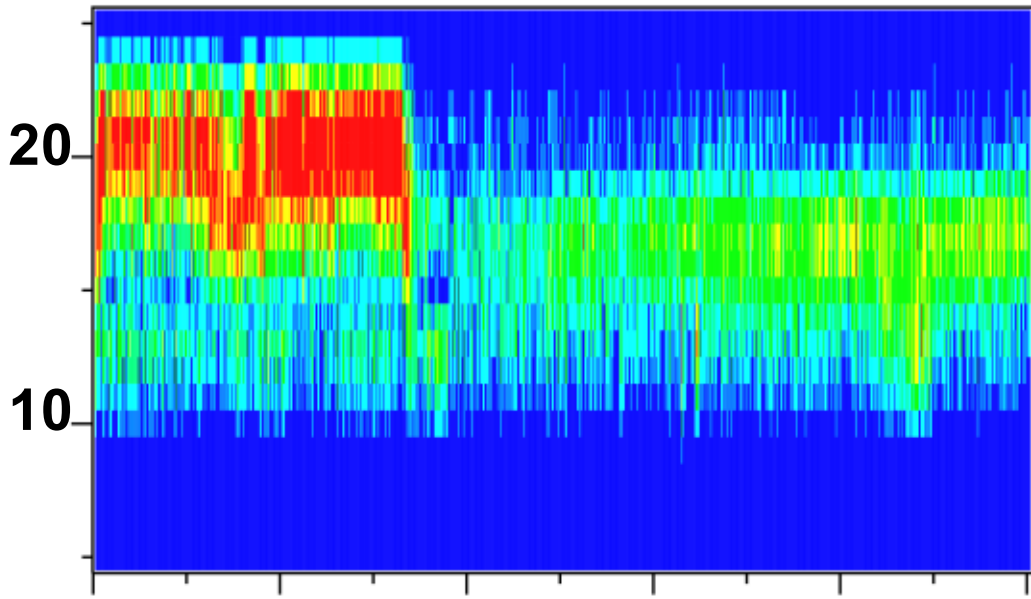

Supplement: Supplementary file 1 [file molecules-26-01250-s001.zip › molecules-1054023-supplementary/figures/tm1_apo_new-eps-converted-to.pdf]

# *h*TSP0-PK11195

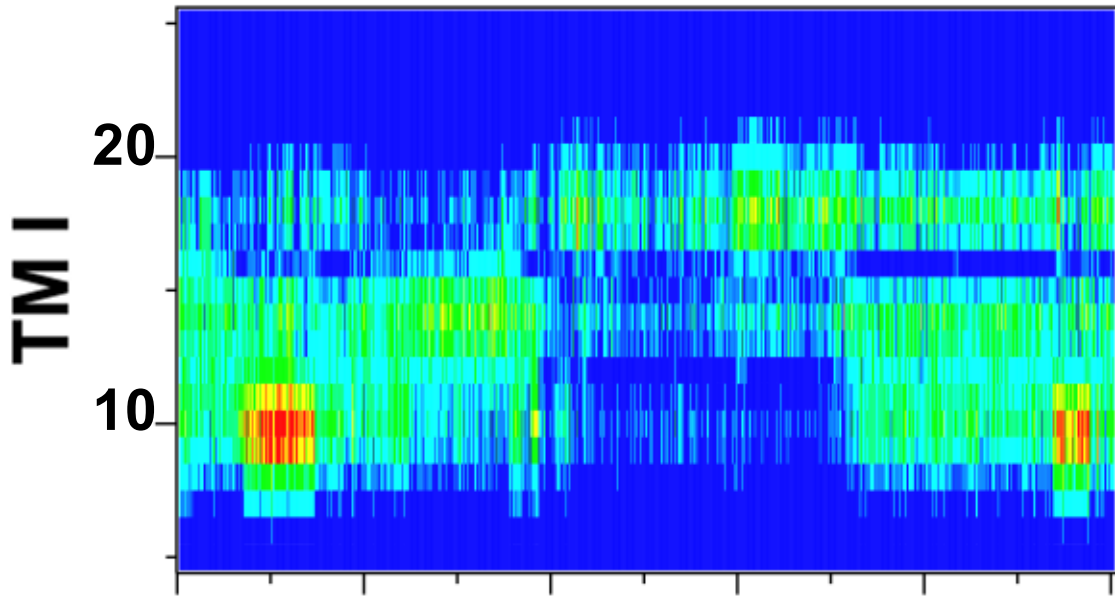

Supplement: Supplementary file 1 [file molecules-26-01250-s001.zip › molecules-1054023-supplementary/figures/tm1_holo_new-eps-converted-to.pdf]

**Residue index**

70

60

50

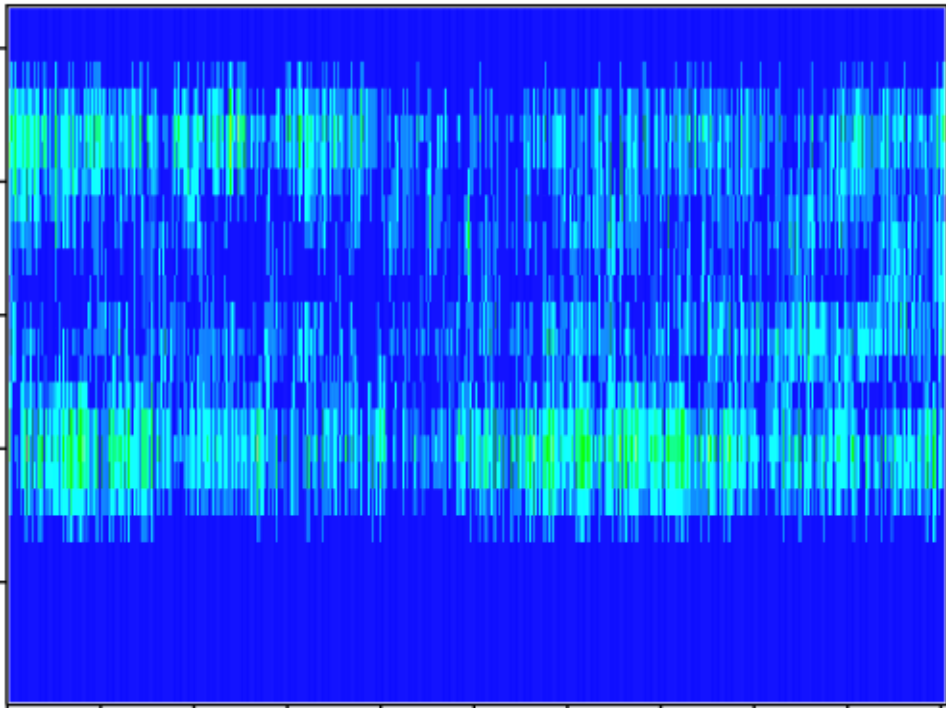

Supplement: Supplementary file 1 [file molecules-26-01250-s001.zip › molecules-1054023-supplementary/figures/tm2_apo_new-eps-converted-to.pdf]

TM II

70

60

50

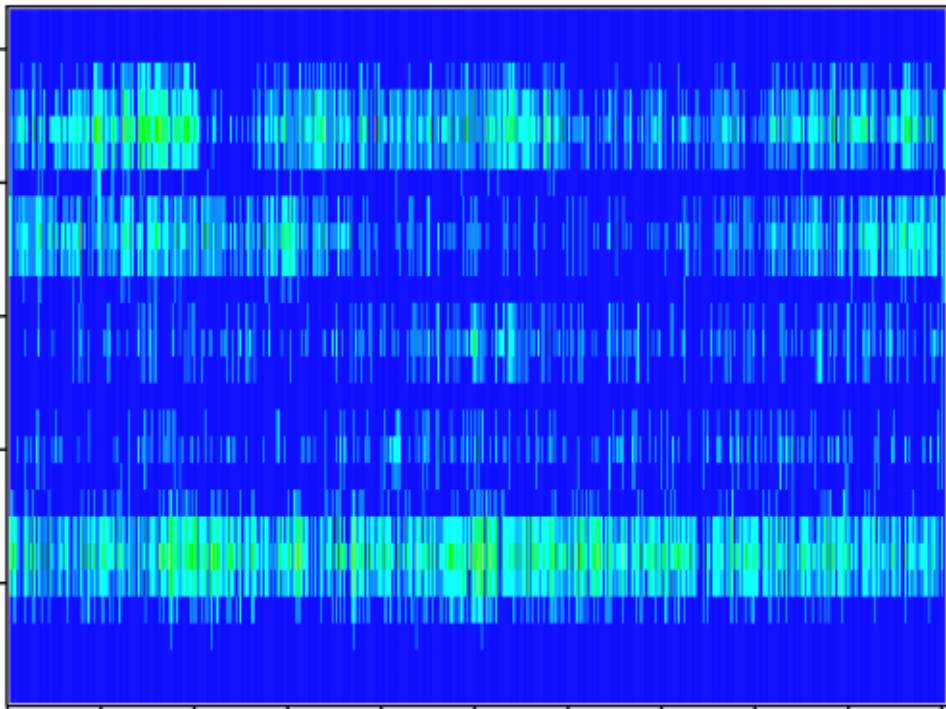

Supplement: Supplementary file 1 [file molecules-26-01250-s001.zip › molecules-1054023-supplementary/figures/tm2_holo_new-eps-converted-to.pdf]

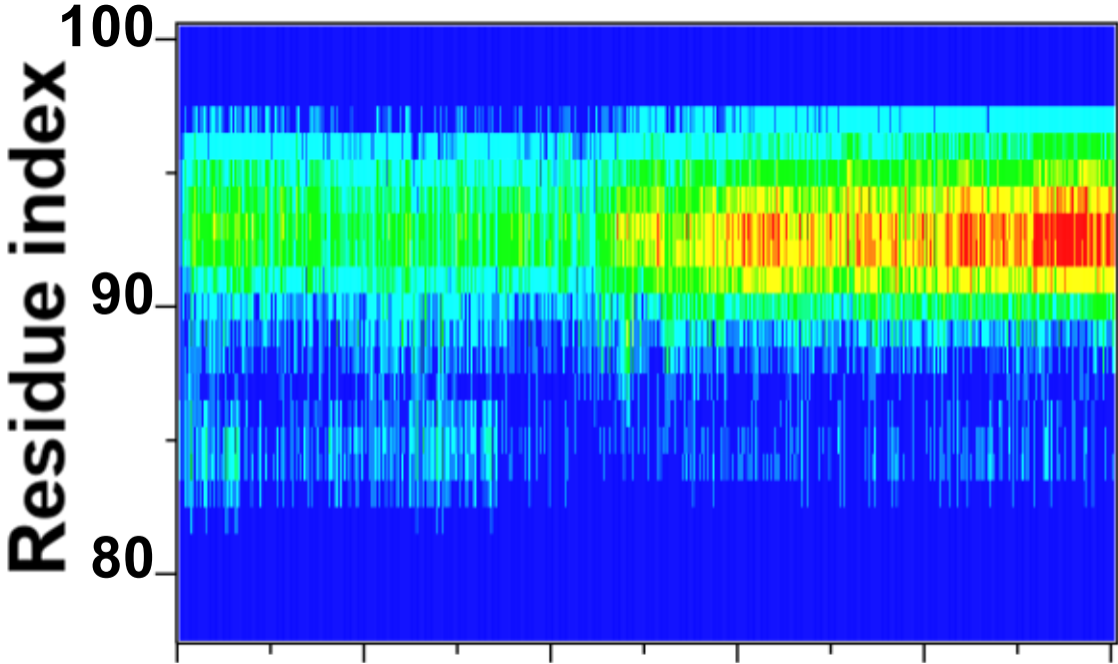

Supplement: Supplementary file 1 [file molecules-26-01250-s001.zip › molecules-1054023-supplementary/figures/tm3_apo_new-eps-converted-to.pdf]

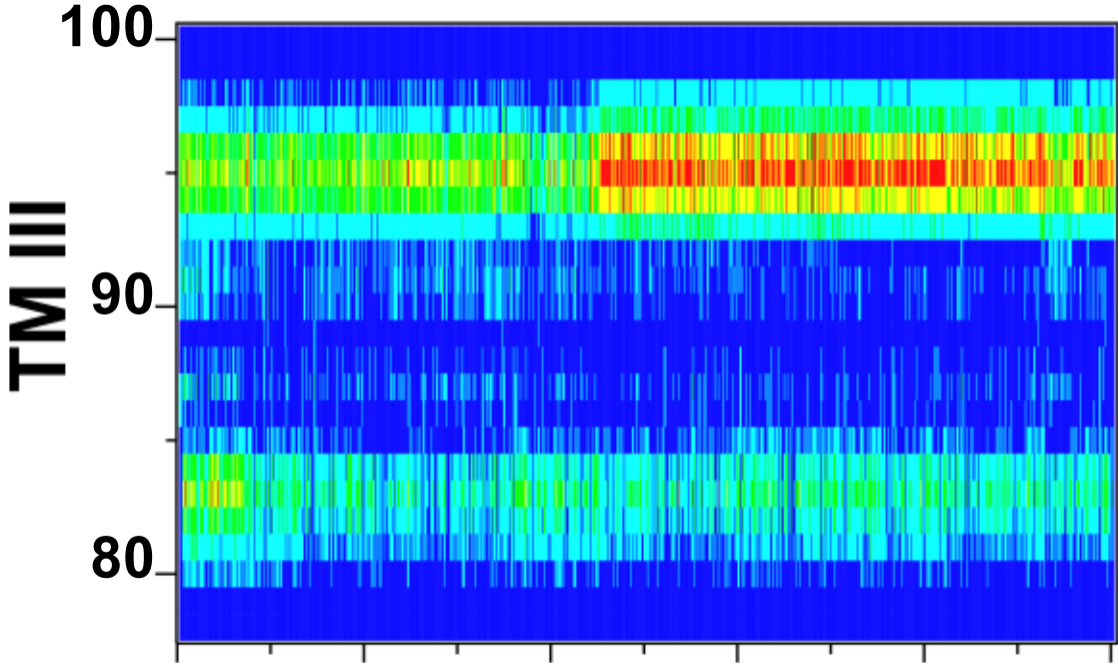

Supplement: Supplementary file 1 [file molecules-26-01250-s001.zip › molecules-1054023-supplementary/figures/tm3_holo_new-eps-converted-to.pdf]

**Residue index**

120

110

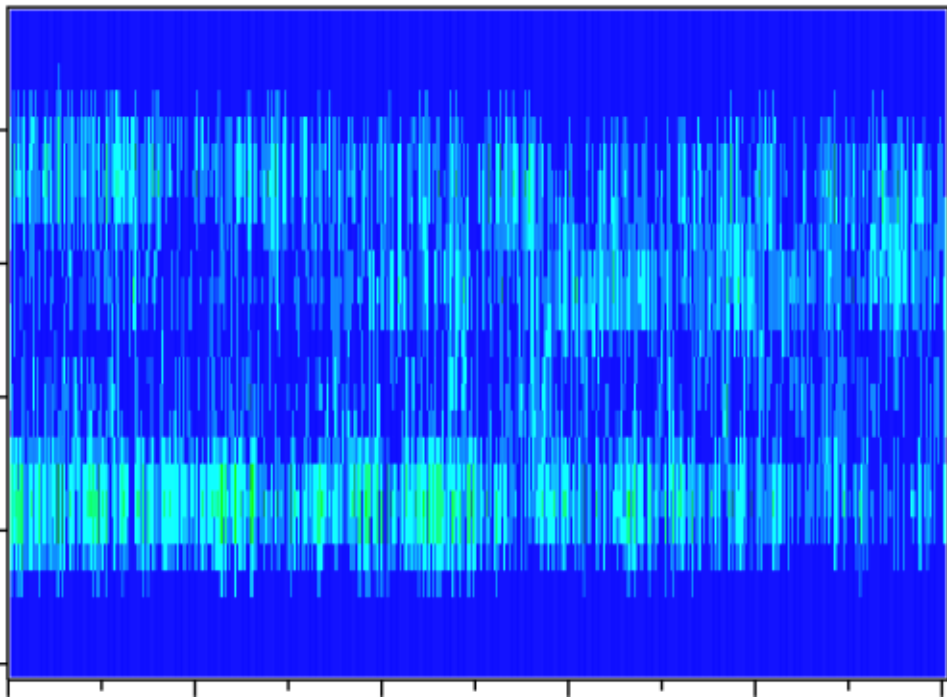

Supplement: Supplementary file 1 [file molecules-26-01250-s001.zip › molecules-1054023-supplementary/figures/tm4_apo_new-eps-converted-to.pdf]

**TM IV**

**120**

**110**

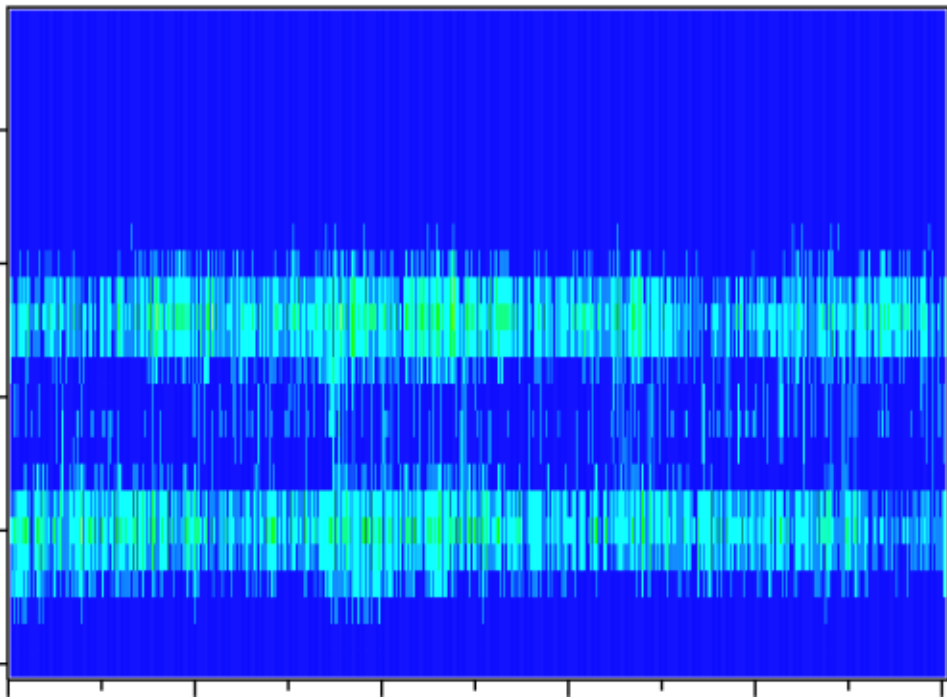

Supplement: Supplementary file 1 [file molecules-26-01250-s001.zip › molecules-1054023-supplementary/figures/tm4_holo_new-eps-converted-to.pdf]

**Residue index**

150

140

0

200

400

600

800

1000

**Time (ns)**

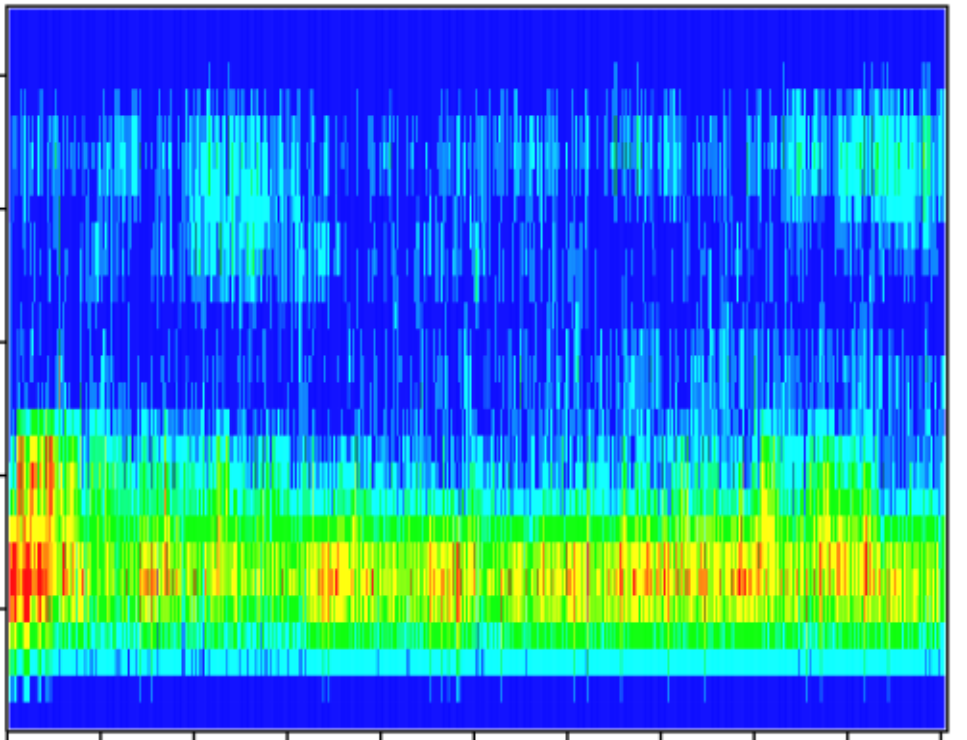

Supplement: Supplementary file 1 [file molecules-26-01250-s001.zip › molecules-1054023-supplementary/figures/tm5_apo_new-eps-converted-to.pdf]

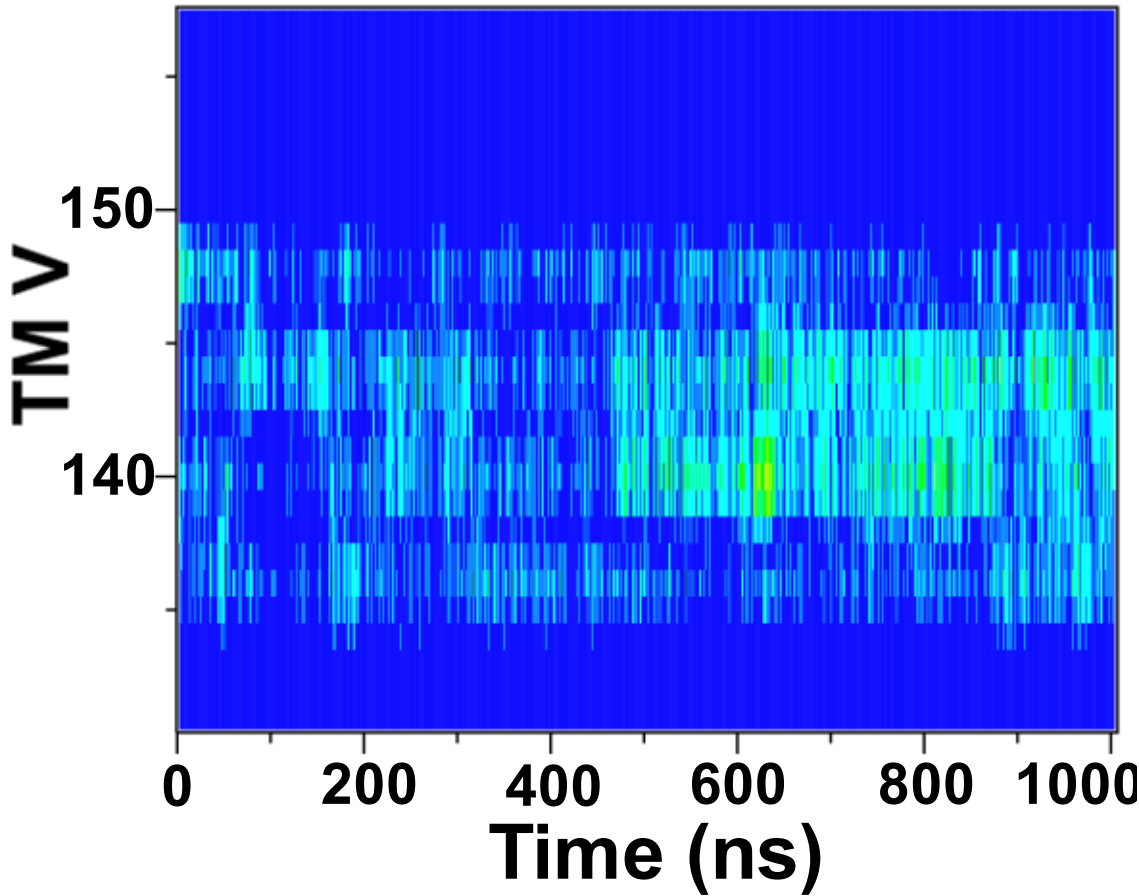

Supplement: Supplementary file 1 [file molecules-26-01250-s001.zip › molecules-1054023-supplementary/figures/tm5_holo_new-eps-converted-to.pdf]
